# Decoding Enzyme–Inhibitor Kinetic Mechanisms by Isothermal Titration Calorimetry: The Case of SARS-CoV‑2 3CL^pro^

**DOI:** 10.1021/acs.analchem.6c00471

**Published:** 2026-06-23

**Authors:** Luca Mazzei, Sofia Ranieri, Davide Silvestri, Gaetano T. Montelione, Stefano Ciurli

**Affiliations:** † Laboratory of Bioinorganic Chemistry, Department of Pharmacy and Biotechnology, 9296 University of Bologna, Bologna I-40127, Italy; ‡ Center for Biotechnology and Interdisciplinary Sciences, 8024 Rensselaer Polytechnic Institute, Troy, New York 12180, United States; § Department of Chemistry and Chemical Biology, Rensselaer Polytechnic Institute, Troy, New York 12180, United States

## Abstract

Accurate kinetic and thermodynamic characterization of enzyme inhibitors remains difficult because conventional activity assays can miss nonequilibrium behavior and provide limited mechanistic resolution. Here, we present an inverse single-injection isothermal titration calorimetry (ITC) workflow that extracts qualitative and quantitative inhibition parameters directly from heat-flow traces by distinguishing rapid reversible, tight-, or slow-binding, and covalent inhibition within a single experimental format. The inhibition of SARS-CoV-2 main protease (3CL^pro^) with ML300, X77, Nirmatrelvir, and Ensitrelvir was used as a benchmark. 3CL^pro^ is pivotal for viral replication, catalyzing polyprotein cleavage into functional nonstructural proteins and representing a key target for structure-based drug design. Despite the development of potent inhibitors, their inhibition mechanisms remain incompletely understood, as conventional assays often lack the resolution to capture the details of enzyme kinetics that are critical for accurate pharmacological characterization and therapy optimization. Model-based fitting of raw calorimetric transients yielded inhibition constants together with mechanistically informative kinetic and thermodynamic parameters without relying on end point readouts. Equilibrium ITC binding measurements on wild-type and mutant 3CL^pro^ variants provided independent validation, revealing distinct affinity and thermodynamic parameters that complemented the inferred inhibitory pathway. These results establish inverse single-injection kinetic ITC as a robust and versatile analytical platform for dissecting complex enzyme inhibition mechanisms, supporting the rational optimization of next-generation SARS-CoV-2 3CL^pro^ inhibitors, and offering broad applicability in drug discovery.

## Graphical abstract

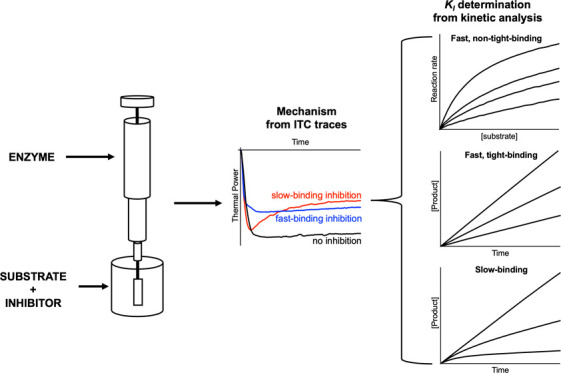

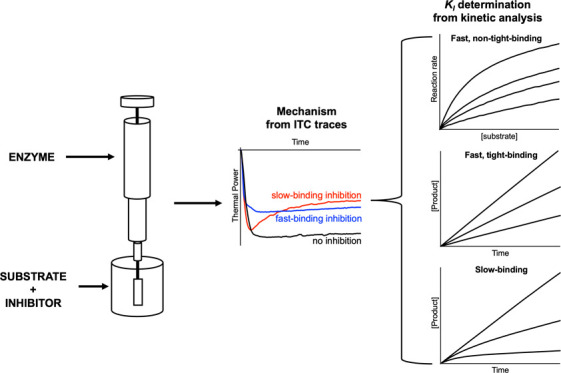

## Introduction

Since its emergence in late 2019, the coronavirus disease 2019 (COVID-19), caused by severe acute respiratory syndrome coronavirus 2 (SARS-CoV-2), has profoundly impacted global health and the economy.^1,2^ In March 2020, the World Health Organization officially declared the outbreak of a pandemic in response to the rapid global spread of COVID-19. Since then, although vaccines and antiviral drugs have markedly mitigated disease severity and transmission, the ongoing evolution of viral variants with altered transmissibility, virulence, and resistance profiles continues to undermine existing therapies. This underscores the urgent need for robust, next-generation antiviral strategies.^3,4^

SARS-CoV-2 ^5^ has a positive-sense, single-stranded RNA genome (∼29.9 kb) that encodes two overlapping polyproteins, pp1a and pp1ab, which are post-translationally cleaved at specific sites by two viral cysteine proteases: the main protease (3CL^pro^ or M^pro^) and the papain-like protease (PL^pro^).^6–8^ The proteolytic activity of these proteases generates a set of nonstructural proteins required for viral replication and host infection.^9–11^

3CL^pro^ (Figure 1) is a ∼68 kDa homodimeric cysteine PA-clan protease.^12^ Each protomer is composed of 306 amino acids and organized in three structural domains.^8,10,13,14^ Domains I (residues 8-101) and II (residues 102-184) adopt a chymotrypsin-like β-barrel fold and harbor the enzyme active site, while domain III (residues 201–306) is involved in the dimerization process and is connected to domain II by a long loop (residues 185-200).

**Figure 1:**
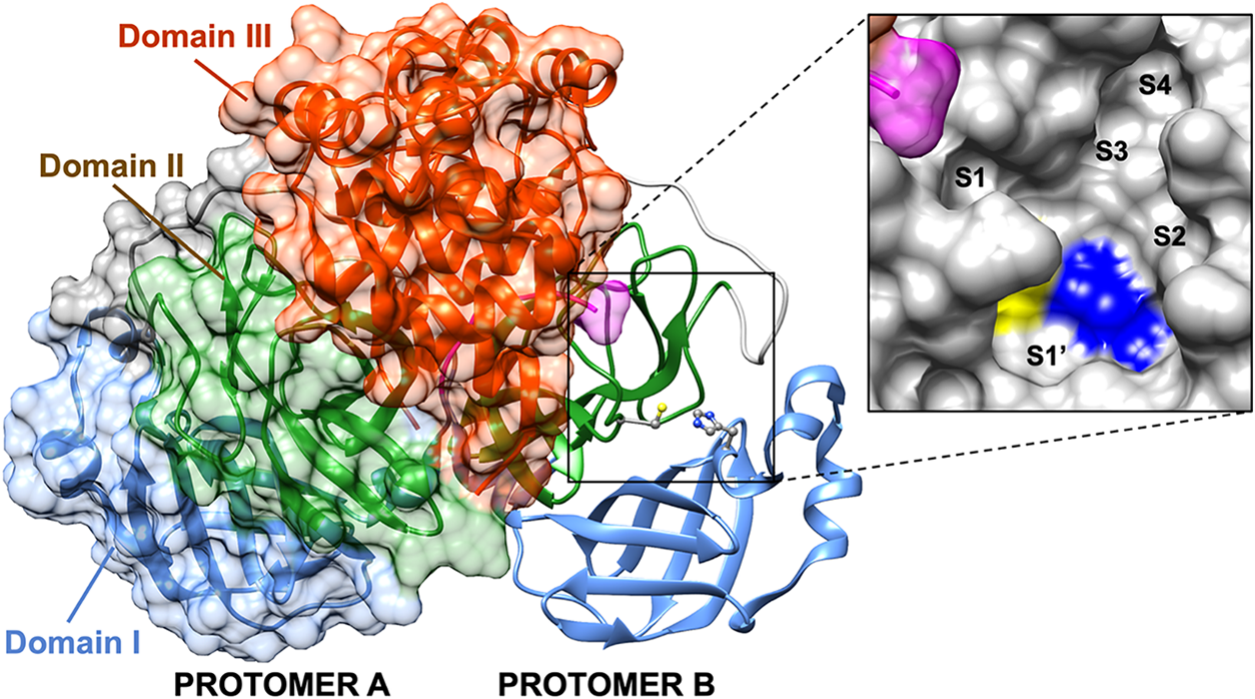
Ribbon representation of the SARS-CoV-2 3CL^pro^ structure (PDB id: 8CDC^14^). Domains I, II, and III are colored in cornflower blue, forest green, and orange-red, respectively. The N-finger region and the loop connecting domains II and III are colored in magenta and gray, respectively. Protomer A is also shown as a surface (transparency 50%). The side chains of the His41–Cys145 catalytic dyad are shown in protomer B as ball-and-stick and colored according to the CPK code. The close-up displays the organization of the subsites in the active site cleft as a surface. Here, surfaces belonging to His41 and Cys145 are colored in blue and yellow, respectively. The N-finger of the adjacent protomer is also shown. Figure made using Chimera^21^ (for interpretation of the references to colors in this figure legend, the reader is referred to the web version of this article).

The active site pocket comprises multiple subsites (S4, S3, S2, S1, and S1′) (Figure 1) that, during hydrolysis catalyzed by the His41–Cys145 catalytic dyad, are occupied by specific sequences of substrate amino acid residues (P4, P3, P2, P1, and P1′, respectively).^10,15^ In particular, a general preference for Leu at P2, a strict requirement for a Gln residue at the P1 position, and Ser or Ala at the P1′ position are observed, with the cleavage site located between P1 and P1′ positions.^10,15^

The reaction catalyzed by 3CL^pro^ follows a two-step mechanism:^13,16^ in a first step, a nucleophilic attack by the thiolate group of Cys145 on the carbonyl C atom of the recognized P1 residue forms an acyl-enzyme intermediate and releases the P1′ fragment of the peptide substrate; in the second step, the acyl-enzyme is hydrolyzed by a nucleophilic attack from an activated water molecule, releasing the P fragment and regenerating the native active site for a next catalytic cycle.

Enzymatic activity is strictly dependent on protease dimerization. Structural and mutagenesis studies have demonstrated that the N-terminal region (called N-finger and comprising residues 1-7) (Figure 1), as well as the residues involved in dimerization at the domain II–III interface, are critical for maintaining dimer stability and structural integrity of the active site pocket.^10,14,17–20^

Given its essential role in viral replication and the lack of homologous proteases in humans, SARS-CoV-2 3CL^pro^ represents a validated target for antiviral drug discovery. Over the past five years, extensive efforts have yielded numerous inhibitors, many derived from earlier campaigns against SARS-CoV-1 or MERS-CoV.^22–27^ These compounds span diverse chemical classes, including peptidomimetics, nonpeptide small molecules, and both synthetic and natural products. A mechanism-based classification system distinguishes the 3CL^pro^ inhibitors discovered so far as either noncovalent or covalent, depending on their binding mode to the enzyme. Noncovalent inhibitors bind to the enzyme through hydrogen bonds and hydrophobic interactions, often employing induced-fit mechanisms to achieve high-affinity binding within the active site. A notable example of this class is Ensitrelvir (S-217622) (Figure 2A), the first orally available, nonpeptidic, noncovalent inhibitor to receive emergency approval in Japan in 2022, developed by Shionogi and sold under the brand name Xocova.^28–30^ Other noncovalent inhibitors include ML300 (Figure 2B) and X77 (Figure 2C), both originally developed against SARS-CoV-1 main protease and later repurposed for the homologous SARS-CoV-2 enzyme.^31–34^ Inhibitors that covalently bind to SARS-CoV-2 3CL^pro^ typically feature a peptidomimetic scaffold incorporating an electrophilic warhead. This inhibition mode usually occurs in two steps: (i) establishment of noncovalent interactions between the enzyme active site pocket and the peptidomimetic backbone, and (ii) nucleophilic attack by the thiol group of the catalytic Cys145 on the electrophilic warhead of the inhibitor, resulting in the formation of a covalent bond that may be reversible or irreversible. Among the numerous covalent inhibitors reported, Nirmatrelvir (PF-07321332) (Figure 2D), developed by Pfizer and marketed (coadministered with ritonavir) as *Paxlovid*,^35,36^ was the first SARS-CoV-2 3CL^pro^ inhibitor to receive regulatory approval, representing a major milestone in antiviral therapy.

**Figure 2:**
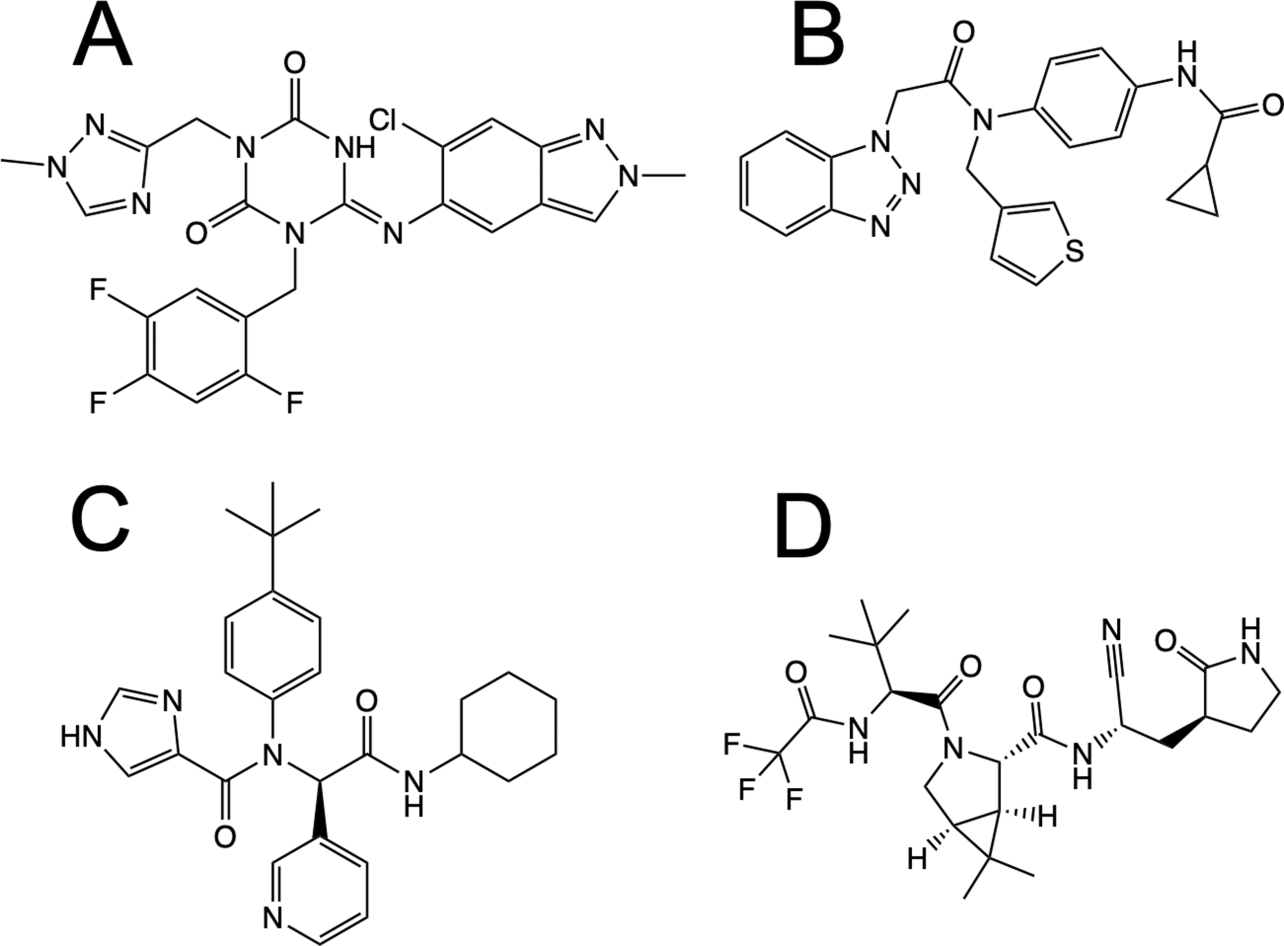
Chemical structures of (A) Ensitrelvir, (B) ML300, (C) X77, and (D) Nirmatrelvir.

Despite the progress in developing potent 3CL^pro^ inhibitors, accurate characterization of their mechanism(s) of inhibition remains a significant challenge. Traditional activity-based assays, such as Förster Resonance Energy Transfer (FRET)^37–40^ and Liquid Chromatography–Mass Spectrometry (LC-MS),^41–43^ are widely used as either continuous or discontinuous assays.^44^ These approaches, however, have several drawbacks and limitations, including the need for customized optically active substrates/products, coupled enzyme reactions, or postreaction separation. Moreover, these methods generally lack the resolution to distinguish between fast- and slow-binding kinetics, reversible and irreversible inhibition, or to describe tight-binding scenarios that deviate from classical Michaelis–Menten kinetics, thus hindering structure–activity relationship studies and rational optimization of inhibitors.

Isothermal titration calorimetry (ITC) has become popular as a robust kinetic method for studying enzyme catalysis and inhibition, as it measures in real time the heat absorbed or released during reactions.^45–49^ This allows virtually any enzymatic reaction to be studied without relying on fluorophores or chromophores as substrates or products, and it does not require postreaction sample separation. In this work, we have developed an ITC-based assay that, exploiting SARS-CoV-2 3CL^pro^ inhibition as a model system, can directly distinguish between reversible fast binding, slow binding, tight binding, and covalent inhibitory molecules. In particular, we extended our initial studies that used the inverse single-injection method to investigate the slow- and tight-binding inhibition of the viral protease by Ensitrelvir^20^ to a representative set of three additional SARS-CoV-2 3CL^pro^ inhibitors with diverse binding mechanisms: ML300, X77, and Nirmatrelvir. These mechanisms were further corroborated by the analysis of the binding thermodynamics of native and two mutated variants of 3CL^pro^. Through these case studies, we demonstrated the capacity of our ITC-based experimental approach to assess inhibition mechanisms, quantify inhibition strengths, and extract thermodynamic parameters, enabling mechanistic classification and providing quantitative parameters that could aid in rational drug design.

## Materials and Methods

### Enzyme, Substrate, and Inhibitor Preparation

Native SARS-CoV-2 3CL^pro^ (monomer molar mass = 33.80 kDa) was expressed in *Escherichia coli* BL21­(DE3) cells using the plasmid vector pGTM_COV2_NSP5_004_SUMO (AddGene, ID: 190062) and purified as previously described.^14^ The dimeric C145A single mutant (monomer molar mass = 33.76 kDa) and the monomeric E290A/R298A double mutant (33.65 kDa) were designed on the native construct by DNA synthesis (GenScript, Rijswijk, Netherlands) and purified using the same procedure. Pure samples of 3CL^pro^ were stored as 0.3 mM aliquots (protein concentration refers to the monomer) at −80 °C in 20 mM Tris-HCl buffer, 50 mM NaCl, 1 mM EDTA, at pH 7.5 (ITC buffer) and diluted to the working concentration using the same buffer prior to each experiment. The peptide substrate WKTSAVLQ↓SGFRKMEW (1.95 kDa; GenScript) was supplied as lyophilized powder and freshly dissolved in the ITC buffer before each experiment. Enzyme and substrate concentrations were determined using molar extinction coefficients (ε_280_) of 32,890 and 11,000 M^–1^ cm^–1^, respectively, as estimated using ProtParam.^50^

Ensitrelvir, X77, and Nirmatrelvir were purchased from MedChemExpress (Sollentuna, Sweden). ML300 was provided by the Cleveland Clinic’s Center for Therapeutics Discovery (Cleveland, OH, USA). All inhibitors, delivered as powders, were dissolved in DMSO at 10 mM, aliquoted (10 μL), and stored at −80 °C. The working solutions were prepared by dilution into the ITC buffer immediately before use.

### Calorimetric Analysis of Enzymatic Inhibition

A detailed description of the calorimetric methodology used in this study and an overview of enzyme kinetics and inhibition are available in the Supporting Information (eqs 1-SI to 9-SI and Figures 1-SI to 3-SI). Inhibition studies were carried out using a VP-ITC microcalorimeter (MicroCal LLC, Northampton, MA, USA) equipped with a computer-controlled 310-μL injection microsyringe and a 1.409 mL sample cell. The reference cell contained deionized water. Experiments were conducted at 298 K with stirring at 300 rpm, recording the thermal power (*TP*, μcal s^–1^) every 2 s using high feedback conditions. All of the experiments were performed in ITC buffer containing 2.5% DMSO to ensure complete solubility of the tested inhibitors. This final DMSO concentration also includes the DMSO content arising from the inhibitor stock solution. The entire set of baseline-uncorrected calorimetric raw data for kinetic analysis is shown in Figure 4-SI.

### Inhibition of 3CL^pro^ by ML300

A preliminary set of experiments was carried out using the progress-curve approach, which relies on the measurement of the initial reaction rates to quantify the ability of a test compound to inhibit the target enzyme.^51^ For these experiments, the syringe contained 5.0 μM enzyme solution and the cell contained 0.60 mM substrate solution, in the absence and presence of ML300 at a concentration of 0.8 or 8 μM. Single injections of enzyme solution (15 μL over 15 s, final enzyme concentration [*E*] = 50 nM) were performed in the cell. Under these conditions, substrate concentration [*S*] largely exceeded *K*_M_ (<100 μM^20^), ensuring substrate-saturating conditions throughout, which is a requirement for this method. *TP* was recorded for 600 s. A baseline correction was applied by fitting the traces recorded in the preinjection period (*ca*. 5 min) of each experiment to an exponential function and subtracting the calculated curves from the calorimetric raw *TP* data over the entire duration of each experiment to obtain baseline-corrected thermograms. The baseline-corrected thermograms were integrated over time starting from the minimum point of the recorded trace, the latter invariably occurring after the nominal response time of the VP-ITC.^20^ The resulting total heat (μcal) was converted to product concentration [*P*] (μM) using the experimentally determined Δ*H*_app_ = −2.0 kcal mol^–1^ for the hydrolytic decomposition of the substrate.^20^ Plotting [*P*] *vs*. time yielded progress curves. The reaction rate of 3CL^pro^ in the absence of ML300 (*v*_0_) was derived from the slope of the corresponding linear progress curve. Initial rates (*v*_*i*_) at 0.8 and 8 μM ML300 were obtained from the slope of the linear region of the corresponding progress curves and normalized to the uninhibited rate (*v*_0_).

Following these preliminary experiments, a Michaelis–Menten approach^20^ was carried out. The syringe was filled with 15 μM enzyme solution, while the cell contained 0.35 mM substrate in the absence or in the presence of increasing concentrations of ML300 (12.5, 25.0, 50.0 μM). Single injections of enzyme solution (20 μL over 20 s; final concentration [*E*] = 0.22 μM) were made into the reaction cell, and *TP* was recorded for variable time periods (1500–4000 s), ensuring that the signal returned to baseline after substrate hydrolysis. *TP* *vs*. time data were processed with Origin 7.0 software (MicroCal) to calculate reaction rates as a function of substrate concentration (according to eqs 1-SI to 5-SI). The data obtained in the absence of inhibitor were fitted to the Michaelis–Menten equation (eq 6-SI) to determine the kinetic parameters *K*_M_ and *k*_cat_. The values of these constants were then used to globally fit all data sets using eq 7-SI to determine the inhibition constant (*K*_I_) and the α parameter, which indicates the inhibition mode (competitive, uncompetitive, or noncompetitive) of ML300.

### Inhibition of 3CL^pro^ by X77

In analogy with the approach used in the case of ML300, the characterization of 3CL^pro^ inhibition by X77 was carried out following the progress-curve approach ([*S*] = 0.60 mM and [*E*] = 50 nM) using 0.10–6.4 μM X77. *TP* was recorded for 600 s, and the same baseline correction procedure, as well as data processing of the baseline-corrected thermograms, was carried out to obtain [*P*] *vs.* time progress curves. Initial rates (*v*_*i*_) at each tested X77 concentration were obtained from the slope of the linear region of the progress curves and normalized to the uninhibited rate (*v*_0_); the values of *v*_*i*_/*v*_0_ were used to derive the apparent inhibition constant (*K*_I_^app^) using eq 8-SI, which was then converted to the true constant *K*_I_ using eq 9-SI, assuming competitive inhibition.

Following these preliminary experiments, a Michaelis–Menten approach^20^ was additionally carried out. The syringe was filled with 15 μM enzyme solution, while the cell contained a 0.35 mM substrate in the absence or in the presence of increasing concentrations of X77 (0.5, 1.0, and 2.5 μM). Single injections of enzyme solution (18 μL over 18 s; final concentration [*E*] = 0.19 μM) were made into the reaction cell, and *TP* was recorded for variable time periods (1500–5000 s), ensuring that the signal returned to baseline after substrate hydrolysis. *TP* *vs.* time data were processed with Origin 7.0 software (MicroCal) to calculate reaction rates as a function of substrate concentration (according to eqs 1-SI to 5-SI). As for ML300 data, the data obtained in the absence of inhibitor were fitted to the Michaelis–Menten equation (eq 6-SI) to determine the kinetic parameters *K*_M_ and *k*_cat_, the latter being used to globally fit all data sets using eq 7-SI in order to determine the inhibition constant (*K*_I_) and the α parameter.

### Inhibition of 3CL^pro^ by Nirmatrelvir

The characterization of 3CL^pro^ inhibition by Nirmatrelvir was carried out following the previously described progress-curve approach using 12.5 nM–0.15 μM Nirmatrelvir. In these cases, *TP* was recorded for 1200 s, and the same baseline correction procedure was applied. The same processing used to obtain ML300 progress curves was applied to baseline-corrected data to yield [*P*] *vs.* time plots. Initial (*v*_*i*_) and steady-state (*v*_*s*_) rates were derived from linear fits of early and late linear segments of the progress curves; the resulting *v*_*i*_/*v*_0_ and *v*_*s*_/*v*_0_ values provided *K*_I_^app^ and *K*_I_^*app^ (eq 8-SI), which were converted to *K*_I_ and *K*_I_^*^ (eq 9-SI) assuming competitive inhibition.

### Calorimetric Analysis of Inhibitor Binding

The binding thermodynamics of native and mutant 3CL^pro^ enzymes to Ensitrelvir, ML300, X77, and Nirmatrelvir were measured using a VP-ITC microcalorimeter (MicroCal LLC, Northampton, MA, USA) equipped with a computer-controlled 310-μL injection microsyringe and a 1.409 mL sample cell. The reference cell contained deionized water. Experiments were conducted at 298 K with stirring at 300 rpm, recording the thermal power (*TP*, μcal s^–1^) every 2 s using high feedback conditions. All of the experiments were performed in ITC buffer containing 2.5% DMSO to ensure complete solubility of the tested inhibitors. This final DMSO concentration also includes the DMSO content arising from the inhibitor stock solution. For Ensitrelvir, X77, and Nirmatrelvir, the syringe contained 300–400 μM ligand solution, while the cell contained 25–40 μM enzyme. Standard titrations were performed with 7-μL injections. For ML300, due to its limited solubility, the syringe contained 300 μM enzyme solution and the cell 40 μM ligand, and an inverse titration was performed with 7-μL enzyme injections. Due to the high affinity of Ensitrelvir and Nirmatrelvir, additional titrations were carried out using 3–5 μM enzyme and 50–150 μM ligand to obtain more reliable *K*_D_ values, thus reducing the c-value. Injection intervals (180–240 s) ensured return to baseline between additions. Control experiments confirmed negligible dilution heats.

*TP* traces were integrated using Origin 7.0 software (MicroCal), and the binding isotherms were fitted by nonlinear least-squares minimization to a single-site model, yielding enthalpy changes (Δ*H*, cal mol^–1^), binding constants (*K*_B_, M^–1^), and stoichiometries (*n*). The value of χ^2^ was used to establish the best fit. The Gibbs free energy *(*Δ*G*) was obtained from Δ*G* = −*RT* ln *K*_B_, and entropy (Δ*S*) from Δ*G* = Δ*H* – TΔ*S*. The reported Δ*H* and Δ*S* values are apparent, reflecting not only binding but also protonation/deprotonation and buffer ionization events. Indeed, the primary goals of the present study were to determine the dissociation constant (*K*_D_ = *K*_B_^–1^) and binding stoichiometry for native and mutant 3CL^pro^ under the same experimental conditions for different inhibitor molecules. The entire set of baseline-uncorrected calorimetric raw data for binding analysis is shown in Figure 5-SI. The complete set of thermodynamic parameters is reported in Table 1-SI. In addition, the calorimetric data sets were independently analyzed using the browser-based ACI-ITC tool (https://aci.sci.yorku.ca/Home/ITC) following the recommended protocol.^52^

## Results

### Calorimetric Studies on the Enzymatic Inhibition of 3CL^pro^

The inhibition of native 3CL^pro^ by ML300, X77, and Nirmatrelvir was analyzed by ITC using the inverse single-injection method. In all instances, the baseline-corrected raw calorimetric traces (Figure 3A,B,C) showed an initial drop in thermal power (*TP*) following enzyme injection, marking the onset of substrate hydrolysis. *TP* reached a minimum within 40–100 s and then evolved in distinct ways depending on the inhibitor tested, indicating different modes of inhibition. Because each inhibitor required specific data treatment, the results are described separately below.

**Figure 3:**
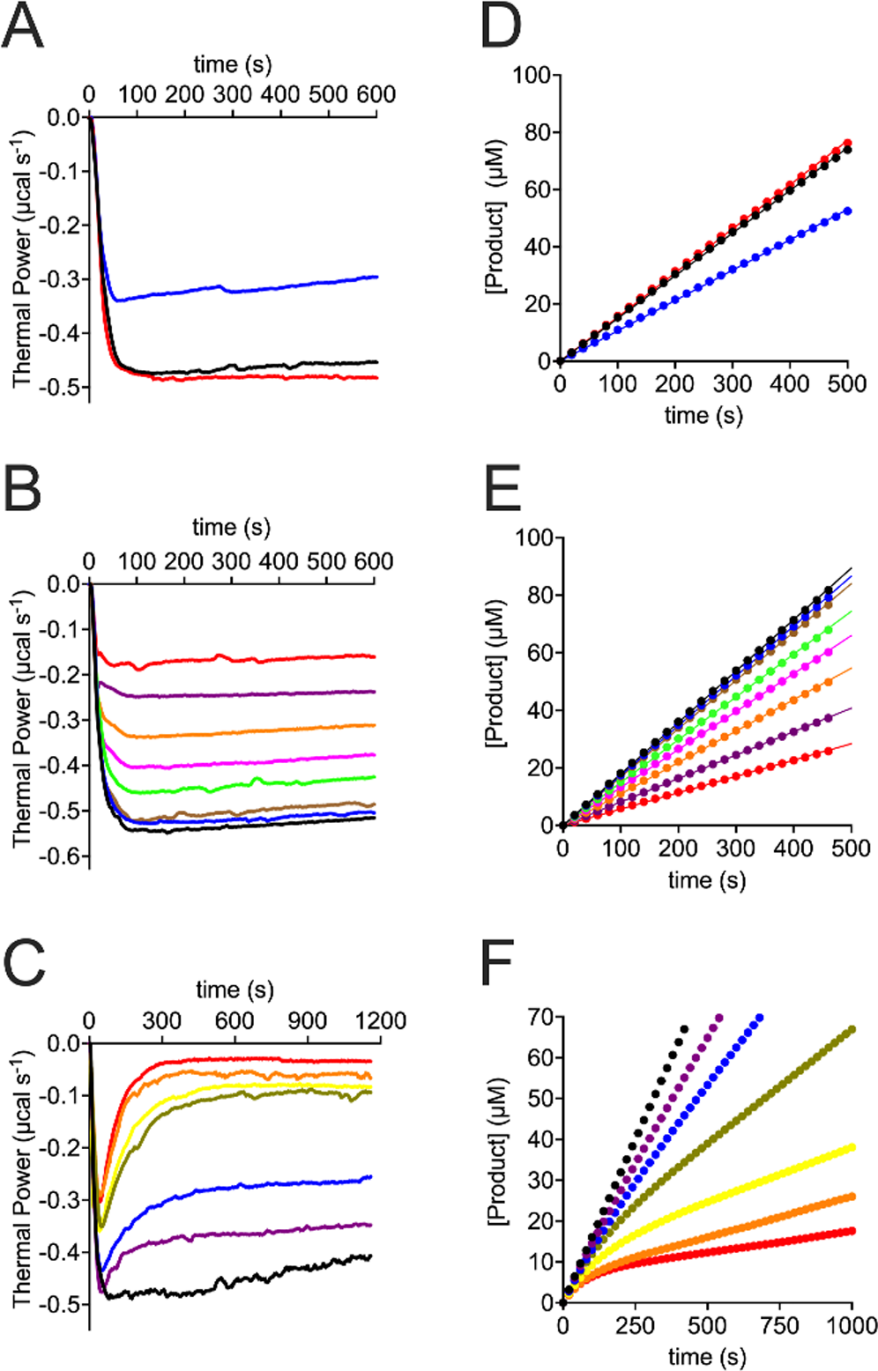
Inhibition of 3CL^pro^ by ML300, X77, and Nirmatrelvir characterized by the ITC–progress-curve approach. (A, B, C) Thermal power traces obtained after injection of 50 nM 3CL^pro^ into the sample cell containing 0.60 mM substrate, either without inhibitor (black) or in the presence of (A) ML300, at concentrations of 0.8 μM (red) or 8 μM (blue), (B) X77, at concentrations of 0.10 μM (blue), 0.20 μM (brown), 0.40 μM (green), 0.80 μM (magenta), 1.6 μM (orange), 3.2 μM (purple), 6.4 μM (red), and (C) Nirmatrelvir, at concentrations of 12.5 nM (purple), 25 nM (blue), 50 nM (green), 75 nM (yellow), 0.10 μM (orange), 0.15 μM (red). (D, E, F) Corresponding reaction progress curves derived from the data in panels A, B, and C. Circles represent a subset of the experimental data, selected every 20 s for clarity.

### Inhibition of 3CL^pro^ by ML300

ML300 is a noncovalent inhibitor of 3CL^pro^,^31,32^ as also shown by X-ray crystallography (PDB id: 7LME).^32^ Previous studies have reported *IC*_50_ values in the low micromolar range,^32,53^ indicating a relatively weak binding, but no inhibition constant *K*_I_ has been experimentally determined.

To define an experimental window suitable for inhibition measurements, ML300 at a concentration of 0.80 μM (inhibitor-to-enzyme molar ratio 16:1) was preliminarily tested using the progress-curves approach, and no inhibition was detectable (Figure 3A). The raw calorimetric trace reached a *TP* minimum (−0.45 μcal s^–1^) approximately 100 s after enzyme injection and remained at a largely stable *TP* level throughout the duration of the experiment, essentially reproducing the curve observed in the absence of inhibitor. The relative progress curve was linear and corresponding to that obtained in the absence of inhibitor (Figure 3D), indicating that ML300 does not inhibit the enzyme at 0.80 μM. Increasing ML300 concentration to 8 μM (inhibitor-to-enzyme molar ratio 160:1) resulted in a largely stable *TP* trace showing a *ca*. 30% reduction in depth (−0.33 μcal s^–1^) compared to the control. The stability of *TP* traces and the linearity of the resulting progress curves were consistent with a fast-binding, reversible inhibition mechanism. The absence of detectable inhibition at moderate inhibitor-to-enzyme ratios and the need for a large excess of inhibitor to observe measurable activity reduction indicated negligible inhibitor depletion upon enzyme binding, thus excluding *a priori* a tight-binding behavior, and enzyme inhibition by ML300 was subsequently characterized using the classical Michaelis–Menten approach. To this aim, inverse single-injection experiments were performed using higher enzyme and lower substrate concentrations (Figure 4A). In the absence of inhibitor, *TP* deflected to approximately −2.1 μcal s^–1^, indicating rapid hydrolysis of the concentrated substrate immediately after enzyme injection, returning to baseline within *ca*. 1000 s; this is consistent with complete substrate consumption. The increase of ML300 concentration (in the range 12.5–50.0 μM) caused a progressive decrease in *TP* minima and slower substrate consumption. The rates *vs.* substrate concentration curves (Figure 4C), derived from the integration of the raw data at each ML300 concentration, followed Michaelis–Menten kinetics. Fitting of the noninhibited data set (eq 6-SI) yielded *K*_M_ = 95 ± 5 μM and *k*_cat_ = 4.0 ± 0.2 s^–1^. Global fitting of all data sets using the complete form of the Michaelis–Menten equation (eq 7-SI) yielded *K*_I_ = 6.2 ± 0.4 μM and α = 17 ± 3, indicating a predominantly competitive inhibition mode.

**Figure 4:**
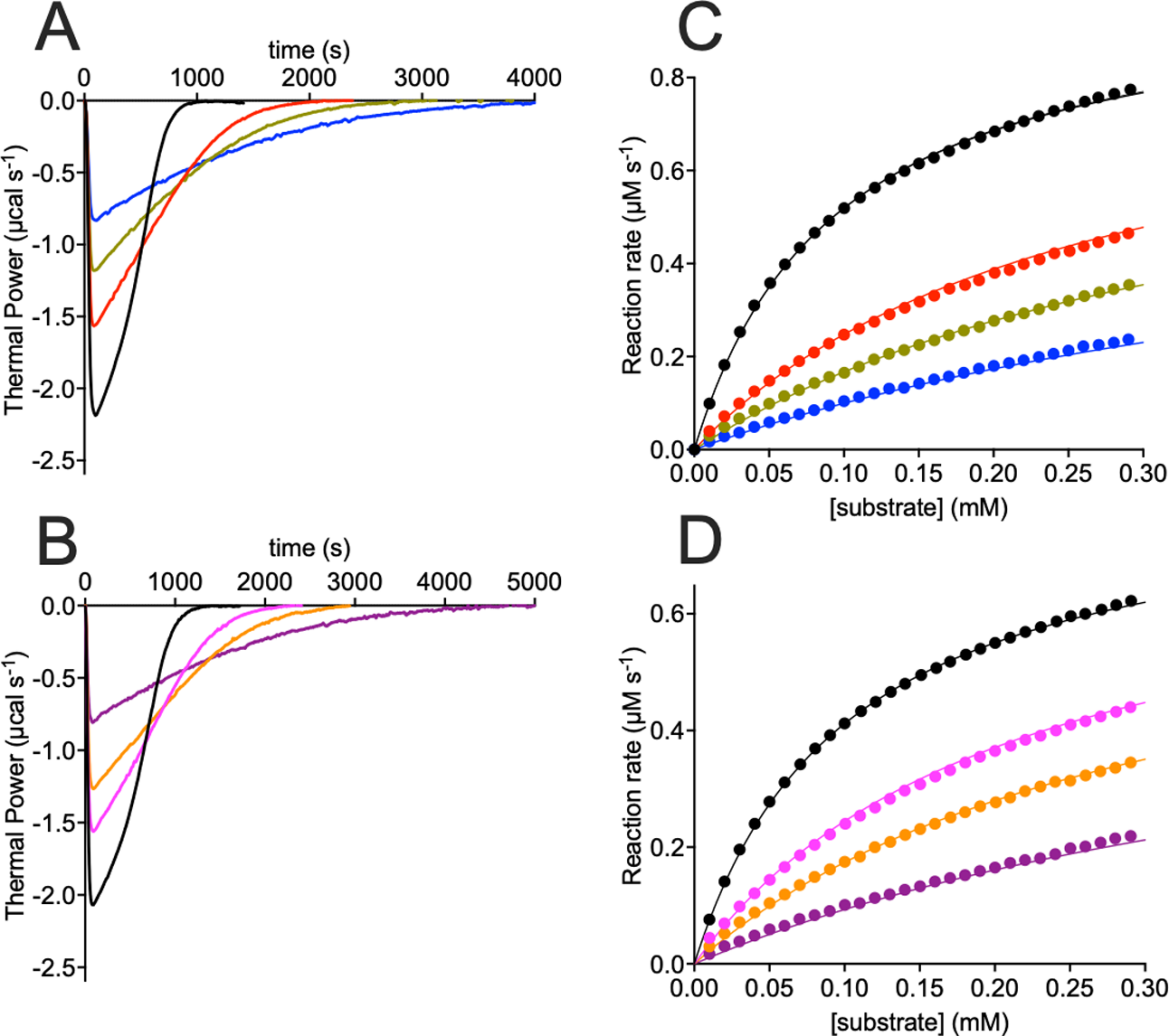
Inhibition of 3CL^pro^ by ML300 and X77characterized by ITC – Michaelis–Menten approach. (A) Thermal power traces obtained after injection of 0.22 μM 3CL^pro^ into the sample cell containing a 0.35 mM substrate, either without inhibitor (black) or in the presence of ML300 at concentrations of 12.5 μM (red), 25 μM (green), and 50 μM (blue). (B) Thermal power traces obtained after injection of 0.19 μM 3CL^pro^ into the sample cell containing 0.35 mM substrate, either without inhibitor (black) or in the presence of X77 at concentrations of 0.5 μM (magenta), 1.0 μM (orange), and 2.5 μM (purple). (C, D) Reaction rates as a function of substrate concentration at increasing concentrations of ML300 (C) and X77 (D) determined from the traces in panels A and B (the color-code is maintained). Data points are shown as circles (subset displayed for clarity, one every 10 μM substrate), with best-fit curves to the Michaelis–Menten model (eqs 6-SI and 7-SI) shown as solid lines.

### Inhibition of 3CL^pro^ by X77

X77 was originally described as a potent noncovalent inhibitor of SARS-CoV 3CL^pro^ (*IC*_50_ = 3.4 μM)^54^ and later repurposed for the homologous SARS-CoV-2 enzyme (*IC*_50_ = 4.1 μM).^33^ The only reported value for the dissociation constant of X77 (0.057 μM) was estimated by computational analysis and not supported by experimental data.^55^ Considering that this value is very small and comparable to the enzyme concentration used in our assays, we first hypothesized tight-binding behavior under these experimental conditions. Accordingly, X77 was treated operationally as a putative tight-binding inhibitor, and the progress-curve approach was employed. Using the inverse single-injection method conducted at substrate-saturating conditions, X77 was initially tested at 0.80 μM (inhibitor-to-enzyme molar ratio 16:1), as previously used for ML300 (Figure 3B). In this case, the raw calorimetric traces reached a *TP* minimum (80 s after enzyme injection) of −0.32 μcal s^–1^, showing a *ca*. 40% reduction with respect to the noninhibited reaction (−0.52 μcal s^–1^), and remained at a largely stable *TP* level throughout the duration of the experiment. This behavior indicated a stronger inhibition than ML300, reinforcing the possibility of a tight-binding behavior. A full characterization of 3CL^pro^ inhibition by X77 was thus carried out, testing inhibitor concentrations ranging from 0.10 to 6.4 μM (Figure 3B). In all cases, raw calorimetric traces exhibited *TP* minima with decreasing depth at higher inhibitor concentrations, indicating a dose-dependent inhibition of enzyme activity. The resulting progress curves were linear (Figure 3E), and reaction rates (*v*_*i*_) decreased with respect to that measured in the absence of the inhibitor (*v*_0_) with increasing concentrations of X77, reaching *ca*. 70% reduction at 6.4 μM. As observed for ML300, the stability of traces over time and the linearity of the progress curves supported a reversible, fast-binding inhibition mechanism for X77. The Morrison’s quadratic model^56–58^ (eq 8-SI) was applied to determine an apparent inhibition constant *K*_I_^app^ = 2.5 ± 0.1 μM (Figure 5A and Table 1). Assuming competitive inhibition (as indicated by several X-ray structures of the 3CL^pro^-X77 complex (PDB ids: 7PHZ, 8P5A, 8P58)^59^ and using *K*_M_ = 95 μM (as determined in the previous experiments), the true inhibition constant was calculated (using eq 9-SI) as *K*_I_ = 0.36 ± 0.02 μM (Table 1) and the resulting value of *K*_I_^app^/[*E*] ratio is approximately 50. According to Strauss and Goldstein,^51,60^ a tight-binding regime should be considered only in the case of this value being <10, therefore, a complementary Michaelis–Menten approach was pursued, assuming a classical, nontight inhibition mode for X77 (Figure 4B,D) as previously described for ML300. The enzyme activity was tested at inhibitor concentrations in the range 0.5–2.5 μM, and the processed data sets were globally fitted using the complete form of the Michaelis–Menten equation, yielding *K*_I_ = 0.39 ± 0.01 μM and α = 16 ± 3, indicating a predominantly competitive inhibition mode. The value of *K*_I_ estimated using the Michaelis–Menten approach was essentially identical to that obtained using the progress-curve methodology (Table 1).

**Figure 5:**
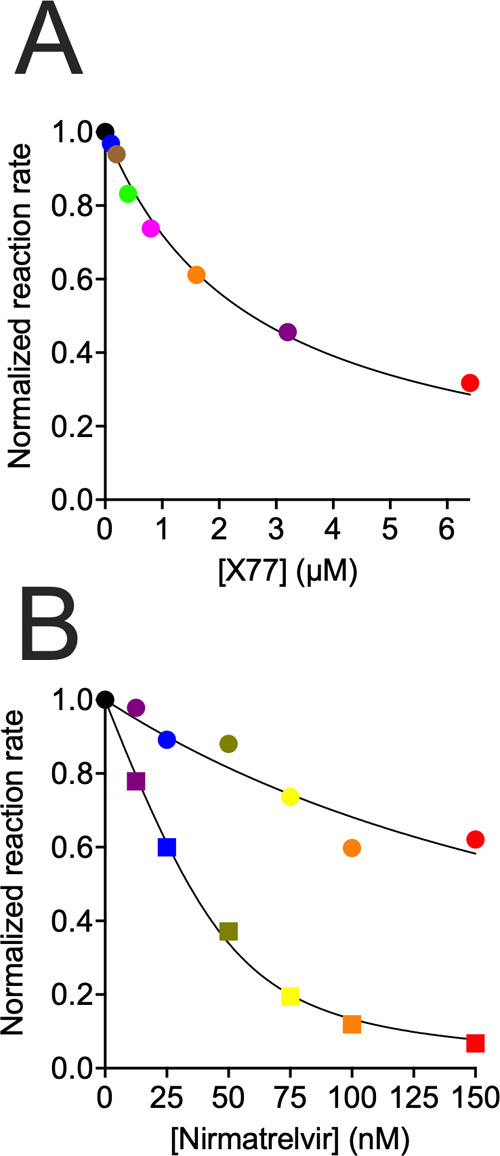
Determination of the inhibition constants of X77 and Nirmatrelvir. (A) Normalized initial reaction rates (*v*_i_/*v*_0_) as a function of X77 concentration with best fits to eq 8-SI. (B) Normalized initial (*v_i_*/*v*_0_, circles) and steady-state (*v*_s_/*v*_0_, squares) reaction rates as a function of Nirmatrelvir concentration with best-fit curves to eq 8-SI.

**Table 1:** ITC-Derived Inhibition and Binding Constants for 3CL^pro^ Inhibitors

	**ML300**	**X77**	**Ensitrelvir** ^20^	**Nirmatrelvir**
	fast	fast	two-step slow-binding	two-step slow-binding
	nontight	nontight	tight	tight
	noncovalent	noncovalent	noncovalent	covalent
***K***_**I**_^**app**^ **(wt)**		2.5 ± 0.1 μM	83 ± 6 nM	180 ± 23 nM
***K***_**I**_ **(wt)**	6.2 ± 0.4 μM^a^	0.36 ± 0.02 μM	9.9 ± 0.7 nM	23 ± 4 nM
		0.39 ± 0.01 μM^a^		
***K***_I_^*app^ **(wt)**			9.5 ± 1.7 nM	8.1 ± 0.8 nM
***K***_I_^*^ **(wt)**			1.1 ± 0.2 nM	1.0 ± 0.2 nM
***K***_**D**_ **(wt)** ^b^	7.5 ± 0.4 μM	2.8 ± 0.3 μM	19 ± 2 nM	15 ± 2 nM
***K***_**D**_ **(wt)** ^c^	3.6 μM (3.3–4.0 μM)	2.2 μM (2.0–2.5 μM)	30 nM (23–38 nM)	20 nM (14–26 nM)
***K***_**D**_ **(C145A)** ^b^			0.15 ± 0.02 μM	4.4 ± 0.8 μM
***K***_**D**_ **(C145A)** ^c^			57 nM (48–67 nM)	3.7 μM (3.3–4.2 μM)
***K***_**D**_ **(E290A/R298A)** ^b^			3.8 ± 0.2 μM	
***K***_**D**_ **(E290A/R298A)** ^c^			1.7 μM (1.6–2.0 μM)	

a Value determined using the Michaelis–Menten approach.

b Value determined using Origin 7.0 and reported as value ± SE.

c Value determined using ACI-ITC and reported as mean value (95% CI).

### Inhibition of 3CL^pro^ by Nirmatrelvir

Nirmatrelvir, developed by Pfizer and marketed (coadministered with ritonavir) under the brand name *Paxlovid* ^35,36^ is a covalent reversible inhibitor of SARS-CoV-2 3CL^pro^.^61^ For Nirmatrelvir, a preliminary inverse single-injection experiment conducted at substrate-saturating conditions in the presence of 0.80 μM inhibitor (inhibitor-to-enzyme molar ratio 16:1) showed no detectable signal, indicating a full abolishment of enzyme activity. This behavior indicated a very strong inhibition, consistent with a tight-binding mechanism.

A full characterization of 3CL^pro^ inhibition by Nirmatrelvir was thus carried out using the progress-curve approach, testing inhibitor concentrations in the range of 12.5 nM–0.15 μM. Unlike ML300 and X77, *TP* traces for Nirmatrelvir (Figure 3C) displayed a characteristic biphasic pattern: an initial concentration-dependent minimum was reached shortly after injection, followed by a gradual rise between 200 and 400 s, before stabilizing at a new plateau. The extent of this increase was also concentration-dependent, and the *TP* traces did not return to preinjection levels even at the highest concentration tested (inhibitor-to-enzyme molar ratio 3:1), suggesting reversibility of the binding. The resulting progress curves (Figure 3F) displayed two distinct phases: an initial linear region transitioning to a slower phase within approximately 400 s. Both the initial (*v*_i_) and the steady-state (*v*_s_) reaction rates, estimated from the slope of the linear portions at early and late phases of each time course, respectively, progressively decreased in a concentration-dependent manner. Consistently with reversible binding, steady-state rates remained >0 and were detectable even at Nirmatrelvir concentrations well above the enzyme concentration. This behavior indicated the presence of a slow-binding inhibition mechanism consisting of two-steps:^62^ (i) a rapid equilibrium is established between the enzyme (*E*), the inhibitor (*I*), and the enzyme–inhibitor (*E*·*I*) complex upon injection of *E* into the sample cell containing both substrate (*S*) and *I*, occurring on a time scale comparable to that of enzyme–substrate (*E*·*S*) complex formation; this initial interaction accounts for the observed decrease in *v*_i_ with increasing Nirmatrelvir concentrations; (ii) a slower rearrangement subsequently occurs to obtain a tighter *E*·I* complex, ultimately leading to the observed steady-state rates *v*_s_. Such a slow-binding mechanism is characteristic of covalent inhibitors exhibiting a reversible mode of binding, confirming the reversibility of the covalent bond between the nitrile group of Nirmatrelvir and the thiol group of the catalytic Cys145 on 3CL^pro^.^35^ Fitting plots of *v*_*i*_/*v*_0_ and *v*_s_/*v*_0_ as a function of inhibitor concentration using eq 8-SI (Figure 5B) yielded apparent inhibition constants *K*_I_^app^ = 180 ± 23 nM and *K*_I_^*app^ *=* 8.1 ± 0.8 nM (Table 1). Assuming competitive inhibition, as suggested by the X-ray crystal structure of the protease–inhibitor complex (PDB id: 8DZ2)^30^ and *K*_M_ = 95 μM, the true inhibition constants were calculated (using eq 9-SI) as *K*_I_ = 23 ± 4 nM (rapid equilibrium) and *K*_I_* = 1.0 ± 0.2 nM (slow equilibrium). Although slow-, tight-binding inhibition has been previously reported for several inhibitors of 3CL-family proteases, including covalent inhibitors of chymotrypsin^63^ and small protein inhibitors of trypsin,^64–66^ Nirmatrelvir has not been previously reported as a slow-binding inhibitor of SARS-CoV-2 3CL^pro^.

### Calorimetric Studies on the Binding of 3CL^pro^ to the Tested Inhibitors

To validate the kinetic inhibition studies, the thermodynamics of 3CL^pro^ binding to ML300, X77, and Nirmatrelvir (in addition to Ensitrelvir, whose kinetics were previously analyzed using the same calorimetric approach^20^) were investigated by ITC experiments performed in the absence of substrate (Figure 6). This strategy aimed at evaluating binding stoichiometries and affinities, and to compare the latter with the inhibition constants determined under catalytic conditions, in the presence of the substrate. For the wild-type enzyme, injection of each ligand into the protein solution generated exothermic peaks (Figure 6A–D), representing the binding of all four molecules to 3CL^pro^. Fitting of the integrated heat data to a single-site binding model (Figure 6E–H) consistently revealed a 1:1 binding stoichiometry per 3CL^pro^ monomer. The resulting dissociation constants (*K*_D_) are reported in Table 1 together with the corresponding errors evaluated using the Origin 7.0 software (MicroCal). The *K*_D_ values obtained for ML300 and X77 were not previously determined and were considered reliable. The *K*_D_ values for Ensitrelvir and Nirmatrelvir are in good agreement with previously reported measurements obtained using the same technique;^67,68^ however, the resulting high c-values (*ca*. 1500) could in principle limit the accuracy of their *K*_D_ estimate.^69,70^ To address this potential limitation, additional titrations were performed at a lower protein concentration, yielding c-values of *ca*. 200. These experiments produced comparable dissociation constants, albeit with a substantially reduced signal-to-noise ratio (Figure 6-SI). Overall, the values of *K*_D_ for all four inhibitors are very similar to those estimated using the kinetic approach, underscoring the reliability of the inverse single-injection ITC method.

**Figure 6:**
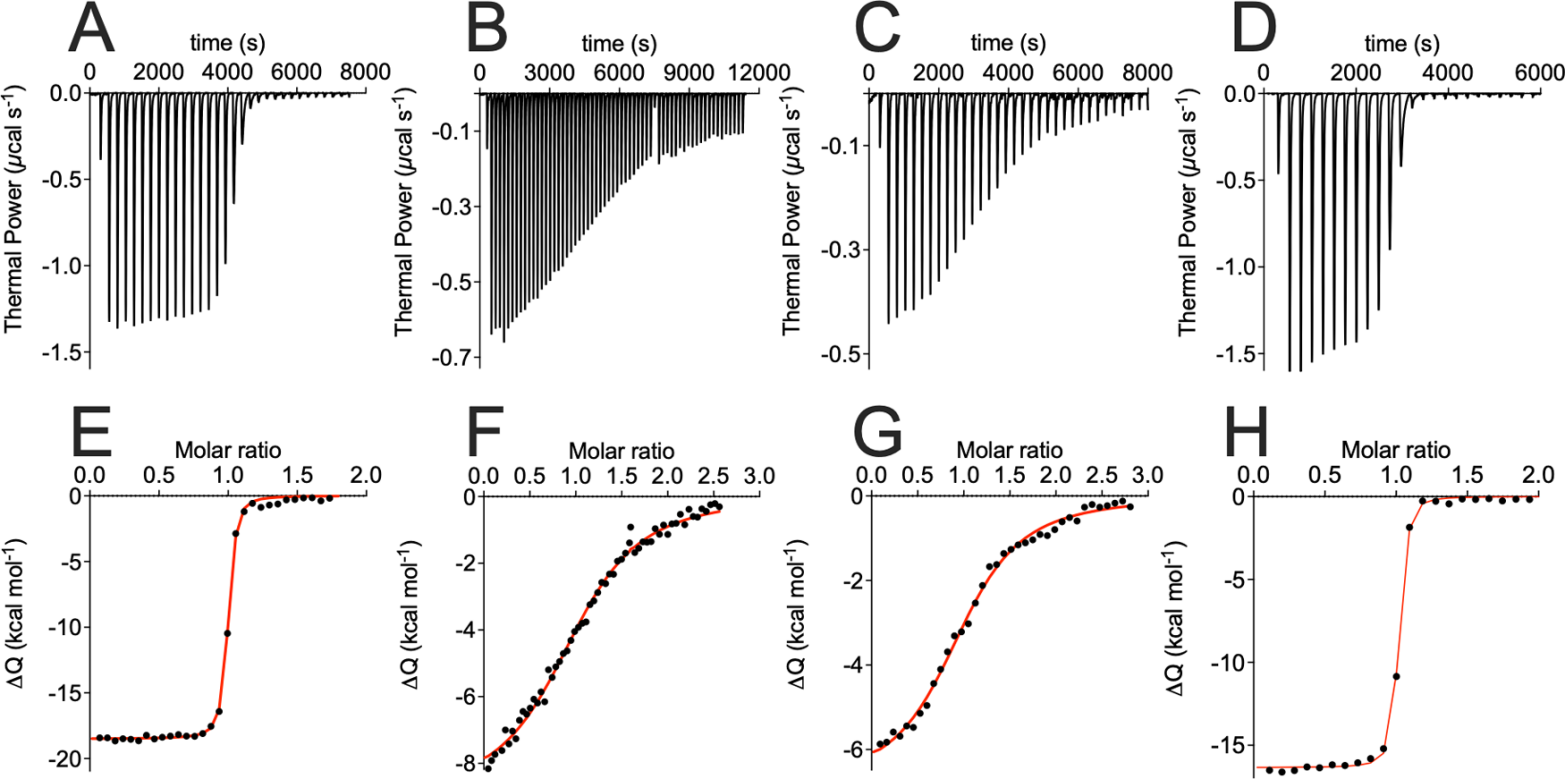
Representative titrations of wild-type 3CL^pro^ with Ensitrelvir, ML300, X77, and Nirmatrelvir. (A, C, D) Heat response for injections of 300 μM Ensitrelvir (A), 300 μM X77 (C), and 400 μM Nirmatrelvir (D) onto 25 μM 3CL^pro^. (B) Heat response for injections of 300 μM 3CL^pro^ onto 40 μM ML300. (E–H) Integrated heats *vs.* molar ratio with best-fit single-site isotherms (red lines).

The effects of active site mutations on the ability of 3CL^pro^ to bind these inhibitors were then investigated (Figure 7). The C145A 3CL^pro^ single mutant, which preserves the dimeric structure of the protease but lacks the catalytic cysteine, still bound Ensitrelvir and Nirmatrelvir, as confirmed by the presence of exothermic peaks upon ligand injection into the protein solution (Figure 7A,B). Data integration and fitting indicated a 1:1 stoichiometry (Figure 7C,D); however, binding affinities were reduced approximately 8-fold and 300-fold, respectively, compared to the wild-type 3CL^pro^ (Table 1), consistent with previously reported calorimetric data.^71^ No measurable heat was detected for ML300 or X77, suggesting that the affinity of these ligands for the enzyme largely decreased in the absence of Cys145, falling below the detection limit of the technique under the experimental conditions used. Consequently, no further characterization was undertaken. X77 has been reported to interact, through its imidazole ring, with the sequence Leu141 -Cys145 of 3CL^pro^.^34^ Mutation of Cys145 to alanine is likely to disrupt this interaction, weakening the binding of X77 to the S1 subsite of 3CL^pro^, which comprises the residues Cys145, His172, Glu166, His163, His164, and Phe140. Similar to X77, the removal of the catalytic cysteine might affect the correct pose of the benzotriazole of ML300 in the S1 pocket,^32^ reducing the binding affinity below the detection limit in the used experimental conditions.

**Figure 7:**
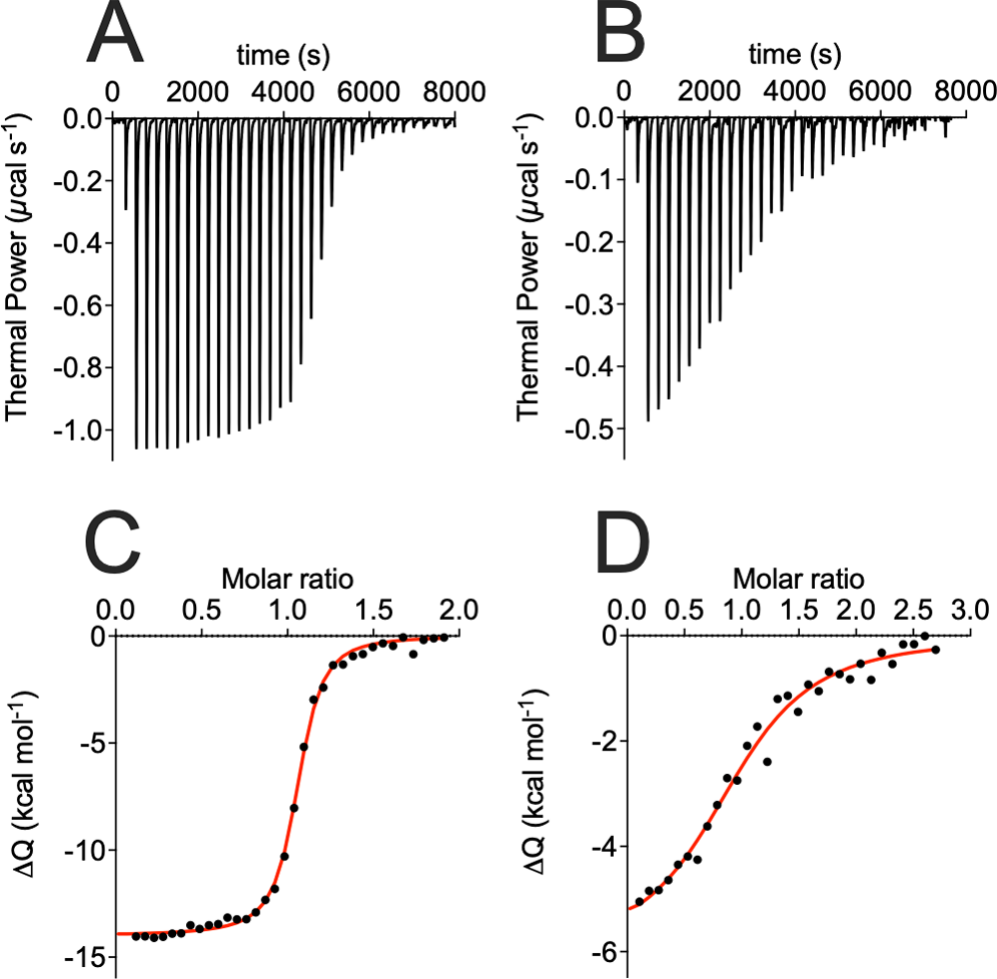
Representative titrations of dimeric C145A 3CL^pro^ with Ensitrelvir and Nirmatrelvir. (A, B) Heat response for injections of 300 μM Ensitrelvir (A) and 400 μM Nirmatrelvir (B) onto 25 μM C145A 3CL^pro^. (C, D) Integrated heats *vs*. molar ratio with best-fit single-site isotherms (red lines).

The decrease of binding affinity for the tested inhibitors was even more pronounced in the case of the E290A/R298A double mutant, which exists in solution as an enzymatically inactive monomer (Figure 8).^18,20^ In this case, binding was only detectable for Ensitrelvir but not for X77, ML300, and Nirmatrelvir (Figure 8A). In the latter case, integration and fitting of the data using a single-site binding model (Figure 8B) indicated a 1:1 stoichiometry and a *K*_D_ corresponding to a nearly 200-fold reduction in affinity relative to the native enzyme and approximately 25-fold weaker binding compared with the dimeric C145A mutant (Table 1). The evident decreased affinity of the monomeric enzyme for either Nirmatrelvir, X77, or ML300 suggested that dimerization is essential to maintain an active-site architecture competent for inhibitor binding. Moreover, the need for dimerization may explain the significant decrease of Nirmatrelvir affinity for the double mutant: although the catalytic cysteine is still present and could, in principle, support covalent binding of the ligand to the catalytic Cys145 thiol, the monomeric state prevents the N-finger belonging to protomer A to maintain structural integrity of the active site pocket of protomer B (and vice versa) (Figure 1). This would severely weaken the interaction between the ligand and the key residues of the protein. Taken together, these results highlight the crucial structural determinants of inhibitor recognition by 3CL^pro^: the mutational analysis underscores the dual importance of the catalytic cysteine and of dimerization in preserving the architecture of the binding pocket, which ensures optimal recognition of these antiviral compounds.

**Figure 8:**
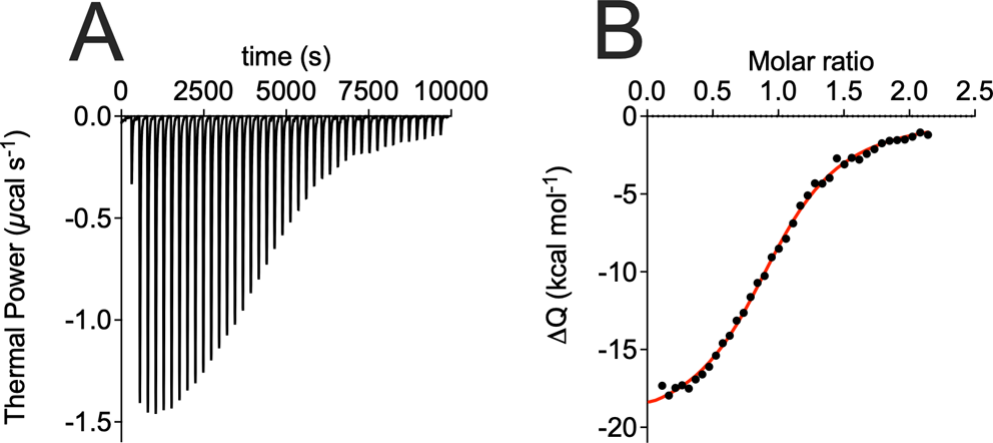
Representative titration of E290A/R298A 3CL^pro^ double mutant with Ensitrelvir. (A) Heat response for injections of 400 μM Ensitrelvir onto 40 μM E290A/R298A 3CL^pro^. (B) Integrated heats *vs*. molar ratio with best-fit single-site isotherm (red lines).

An independent analysis was performed using the ACI-ITC method to account for systematic concentration uncertainties and to provide statistically rigorous confidence intervals for the fitted parameters.^52^ This analysis confirmed the affinity trends obtained from conventional nonlinear fitting while yielding broader confidence intervals, consistent with a more realistic estimation of the parameter uncertainty. The resulting *K*_D_ values and 95% confidence intervals (in brackets) are also reported in Table 1 (and in Figure 6-SI for the additional titrations of Ensitrelvir and Nirmatrelvir performed at a lower protein concentration).

## Discussion

The accurate characterization of inhibition mechanisms and strengths is a crucial challenge in antiviral drug development. Distinguishing the inhibition potency of a ligand, whether it acts through a fast- or slow-binding inhibition mechanism, and whether it binds reversibly or irreversibly, provides critical insights into its pharmacologic behavior and, ultimately, its therapeutic potential. Conventional enzymatic assays, although widely used, often lack the resolution needed to dissect such mechanistic features, particularly when inhibitors display complex behaviors such as tight-binding or slow-binding kinetics. In this context, isothermal titration calorimetry (ITC) offers a uniquely powerful solution. As a direct, label-free technique, ITC not only measures binding affinities but also provides information on the thermodynamic and kinetic parameters of inhibition. Building on our previous work on Ensitrelvir, where we demonstrated the capacity of ITC to reveal slow- and tight-binding inhibition of SARS-CoV-2 3CL^pro^,^20^ the present study extends this approach to additional inhibitors of diverse mechanistic properties. Here, we show that the inverse single-injection ITC method can reliably detect and quantify inhibition by ML300 (fast, weak-binding, noncovalent reversible inhibitor), X77 (fast, intermediate-binding, noncovalent reversible inhibitor), and Nirmatrelvir (covalent, tight-binding, reversible inhibitor). Together with Ensitrelvir, these case studies illustrate how the ITC-based inverse single-injection method can capture a broad spectrum of inhibition modes, providing a powerful and versatile tool for the detailed characterization of inhibition mechanisms. When performed under substrate-saturating conditions, this approach yields linear progress curves in the absence of inhibitors, thereby ensuring that any deviations in enzyme activity can be attributed solely to inhibition. Under these conditions, the raw thermal power trace itself becomes informative: a stable trace following injection indicates rapid attainment of the enzyme–inhibitor equilibrium (fast-binding), whereas a gradually evolving trace reveals the slower establishment of equilibrium (slow-binding). This contrasts with traditional assay protocols, in which inhibitors are preincubated with the enzyme prior to substrate addition,^37–43^ a setup that can mask slow-binding kinetics. The proposed method also accommodates the challenges posed by inhibitors of widely differing affinities. For medium- to low-affinity ligands, which exert significant inhibition at concentrations much larger than that of the enzyme, Michaelis–Menten analysis of initial reaction rates *vs.* substrate concentration allows extracting inhibition constants and defining the inhibition mode. In contrast, for tight-binding inhibitors, where significant inhibition is achieved when inhibitor and enzyme concentrations are comparable, progress-curve analysis that monitors the full-time course of product formation becomes necessary. In this case, one can not only quantify inhibition strength but also distinguish between fast- and slow-binding behavior; in the case of covalent reversible inhibitors, the transition from an initial complex to a more tightly bound covalent state can also be captured. Covalent and noncovalent inhibitors that exhibit slow tight-binding kinetics are particularly interesting for drug development because they can have prolonged drug residence times and sustained pharmacological effects, often providing better pharmacokinetics, pharmacodynamics, and dosage requirements.^72^

**Figure 9:**
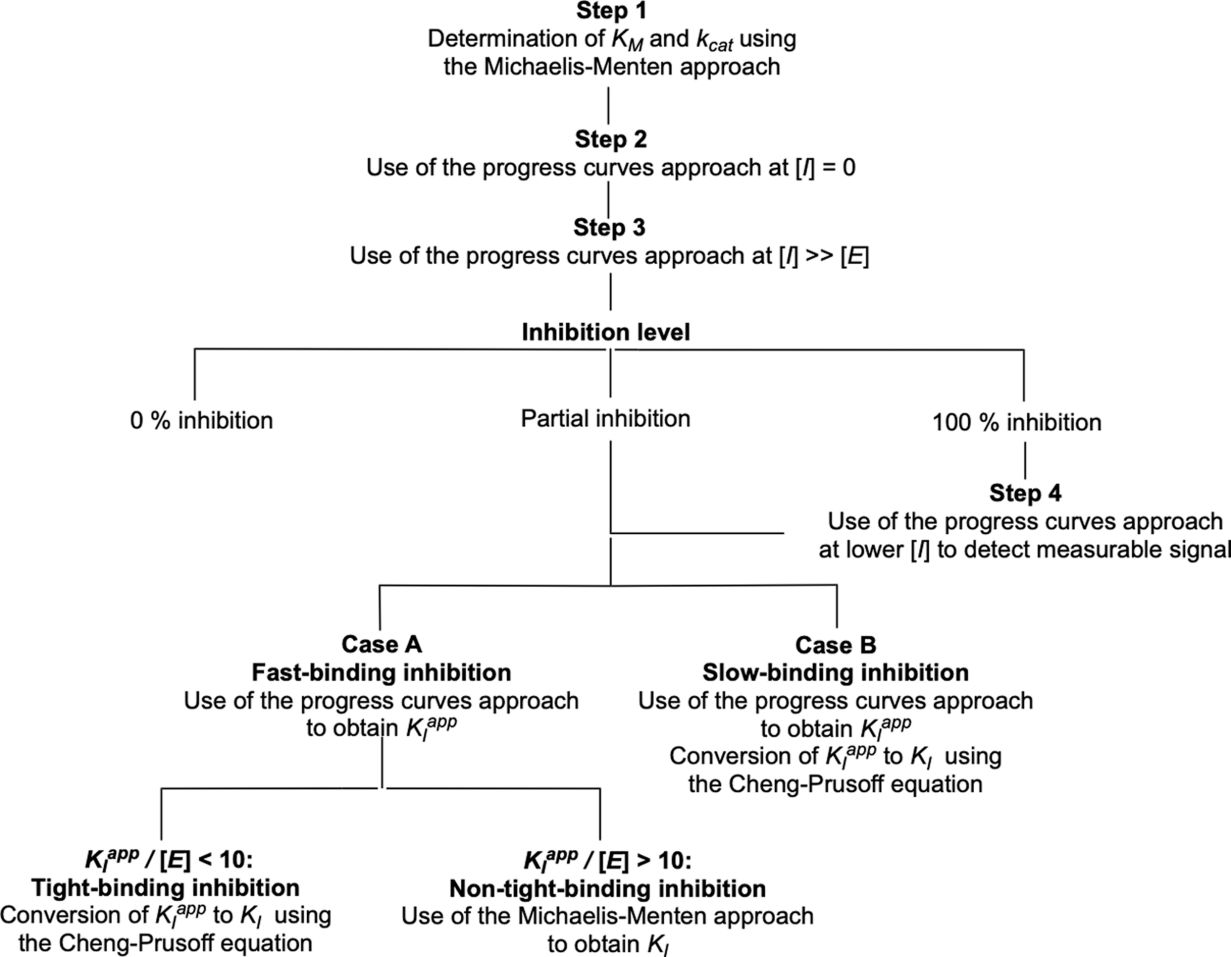
Workflow for characterizing enzyme inhibition mechanisms by ITC using the inverse single-injection method.

The experimental workflow proposed in Figure 9 provides a roadmap for the complete characterization of any enzyme inhibition study by using calorimetry. The kinetic parameters *K*_M_ and *k*_cat_ of the enzyme–substrate pair are initially established using a Michaelis–Menten approach (step 1). Then, an initial inverse single-injection experiment carried out at a substrate concentration [*S*] much greater than the experimentally determined *K*_M_ verifies substrate saturation and steady-state conditions, revealed by the linearity of the progress curve (step 2). The same experiment is then repeated with an inhibitor concentration [*I*] much larger than that of the enzyme [*E*] (step 3), and the resulting thermal power traces guide the choice of the subsequent analytical strategy. The observation of a trace reproducing that recorded in the absence of inhibitor indicates that the latter does not inhibit the enzyme at the tested concentration (0% inhibition); in this case, the inhibitor concentration could be increased to observe detectable inhibition (or to confirm lack of interaction). On the other hand, a trace that reveals a reduction in the thermal power indicates the occurring of partial inhibition, and the shape of the recorded trace distinguishes between two different mechanisms: a trace that is stable over time indicates fast binding (case A), whereas a gradually evolving trace indicates slow-binding (case B). While the slow-binding behavior imposes the use of a progress-curves approach to determine the mechanism and the inhibition constant(s), in case A, the data treatment depends on the affinity of the inhibitor for the enzyme: for tight-binding inhibitors, depletion of the inhibitor due to enzyme binding cannot be neglected, whereas this effect is negligible for nontight-binding inhibitors. Consequently, a conservative analysis based on the progress-curve method should initially be adopted to obtain an estimate of the apparent inhibition constant *K*_I_^app^. This strategy avoids the *a priori* assumption that the total inhibitor concentration equals the free inhibitor concentration, an approximation valid only for nontight-binding inhibitors, enabling detection of deviations from simple steady-state kinetics. The subsequent analytical step is guided by the Strauss and Goldstein criterion:^51,60^ if *K*_I_^app^/[*E*] < 10, the inhibitor is classified as tight-binding, inhibitor depletion cannot be ignored, and the progress-curve analysis must be retained. Conversely, when *K*_I_^app^/[*E*] > 10, ligand depletion by enzyme binding is negligible, the inhibitor is considered nontight-binding, and classical Michaelis–Menten analysis can be applied. If, following Step 3, 100% inhibition is observed, additional inverse single-injection experiments and the progress-curves approach must be used at progressively lower inhibitor concentrations to recover measurable calorimetric signals (Step 4). Also in this case, the stable *vs.* evolving trend of the thermal power traces distinguishes between fast-binding and slow-binding, and data should be treated as previously described for cases A and B.

The values of the inhibition constants determined using the inverse single-injection methodology were validated by measuring the enzyme–inhibitor affinities through ITC binding equilibrium experiments in the absence of substrate. ML300, for which no *K*_D_ had previously been reported, displayed consistent values between binding and kinetic assays (Table 1), as well as with low micromolar *IC*_50_ data.^32,53^ For X77, the thermodynamic *K*_D_ is consistent with the apparent inhibition constant *K*_I_^app^ and within the same order of magnitude as the calculated *K*_I_ value (Table 1). In the case of Ensitrelvir and Nirmatrelvir, the thermodynamic *K*_D_ values are consistent with the inhibition constants *K*_I_ and only slightly larger than *K*_I_^*^ (Table 1). Overall, this double approach confirmed the reliability of the kinetic methodology proposed in this study for the identification of enzymatic inhibition modes by using calorimetry.

## Conclusions

In this study, we expanded the application of the inverse single-injection ITC methodology, recently applied for the characterization of SARS-CoV-2 3CL^pro^ inhibition by Ensitrelvir, to three additional representative viral drugs, namely, ML300, X77, and Nirmatrelvir, which cover a wider range of inhibitory mechanisms, including reversible fast-binding, slow-binding, tight-binding, and covalent inhibition. Our results show that this kinetic calorimetric approach enables not only quantitative determination of inhibition constants but also direct identification of key mechanistic features, including fast-, tight-, and slow-binding behavior directly from the raw calorimetric traces.

The overall agreement between the inhibition constants derived from kinetic analyses and the thermodynamic parameters obtained from equilibrium ITC binding experiments supports the reliability of the ITC-based method in capturing both the energetics and kinetic complexity of enzyme–inhibitor interactions. At the same time, our results highlight the importance of combining complementary kinetic and thermodynamic measurements to obtain a more complete description of enzyme–inhibitor interactions.

Taken together, these findings establish ITC, and particularly the inverse single-injection protocol, as a versatile and highly informative tool for the study of enzyme inhibition. By allowing inhibition potency, binding mechanism, and thermodynamic properties to be examined within a unified experimental framework, this methodology is well-suited for application in drug-discovery workflows. Although demonstrated here on SARS-CoV-2 3CL^pro^, the approach is broadly applicable to other viral and cellular proteases, and more generally to enzyme systems in which mechanistic complexity extends beyond the assumptions of classical Michaelis–Menten analysis.

Overall, this work positions inverse single-injection ITC as a powerful platform for mechanistic enzymology and as a broadly applicable strategy for the quantitative characterization and rational development of small-molecule enzyme inhibitors.

## Supplementary Material



## Data Availability

The calorimetric raw data are available from the corresponding author upon request.

## Notes

General description of the methodology; overview of enzyme kinetics and inhibition; baseline-uncorrected raw calorimetric traces of kinetics and binding experiments; additional ITC binding experiments; and thermodynamic parameters derived from ITC binding experiments (PDF)
