# Supplementary material for: Decoding Enzyme–Inhibitor Kinetic Mechanisms by Isothermal Titration Calorimetry: The Case of SARS-CoV‑2 3CLpro
Source: Anal Chem. 2026 Jun 23;98(26):19520–32. doi: 10.1021/acs.analchem.6c00471 (PMC13347698; doi:10.1021/acs.analchem.6c00471)
Supplement: Supplementary file 1 [file ac6c00471_si_001.pdf]

Decoding Enzyme–Inhibitor Kinetic Mechanisms by Isothermal Titration Calorimetry: The Case of SARS-CoV-2 $3CL^{pro}$

Luca Mazzei,[1,*] *Sofia Ranieri,*[1] Davide Silvestri,[1]

Gaetano T. Montelione,[2,3] *and Stefano Ciurli*[1,*]

[1]Laboratory of Bioinorganic Chemistry, Department of Pharmacy and Biotechnology, University of Bologna, I-40127 Italy

[2]Center for Biotechnology and Interdisciplinary Sciences, Rensselaer Polytechnic Institute, Troy, New York, 12180, USA

[3]Department of Chemistry and Chemical Biology, Rensselaer Polytechnic Institute, Troy, New York, 12180, USA

* Corresponding authors; email: luca.mazzei2@unibo.it; stefano.ciurli@unibo.it

Luca Mazzei	orcid: 0000-0003-1335-9365	e-mail: luca.mazzei2@unibo.it
Sofia Ranieri	orcid: 0009-0001-9046-4903	e-mail: sofia.ranieri2@studio.unibo.it
Davide Silvestri	orcid: 0009-0002-1852-5131	e-mail: davide.silvestri11@studio.unibo.it
Gaetano T. Montelione	orcid: 0000-0002-9440-3059	e-mail: monteg3@rpi.edu
Stefano Ciurli	orcid: 0000-0001-9557-926X	e-mail: stefano.ciurli@unibo.it

SUPPLEMENTARY INFORMATION

General description of the methodology

An isothermal calorimeter consists of two cells, namely a reference cell (usually filled with deionized water) and a sample cell, contained in an adiabatic shield. A computer-controlled microsyringe is mounted on the sample cell, where it dispenses its content using a rotating, paddle-shaped needle that ensures complete mixing of the solutions after each injection. During an ITC experiment, a thermoelectric device continuously measures the temperature difference between the sample and reference cells and, using a cell feedback network, it maintains this difference (ΔT) at zero by adding or removing heat from the sample cell. The amount of heat (*Q*) added or removed by the system over time (*t*) is defined as the thermal power (*TP*) (Eq. 1-SI):

$$TP = \frac{dQ}{dt} \quad \text{Eq. 1-SI}$$

In an enzyme-catalyzed reaction, the heat associated with the conversion of *n* moles of substrate to product at constant pressure is described by Eq. 2-SI:

$$Q = n \cdot \Delta H_{app} = [S] \cdot V_{cell} \cdot \Delta H_{app} \quad \text{Eq. 2-SI}$$

Here, ΔH_{app} is the total apparent molar enthalpy for the reaction, [*S*] is the molar concentration of converted substrate, and V_{cell} is the volume of the sample cell where the reaction occurs. The reaction rate, defined as the change in substrate concentration over time, can be related to the thermal power by Eq. 3-SI:

$$v = -\frac{d[S]}{dt} = \frac{1}{V_{cell} \cdot \Delta H_{app}} \frac{dQ}{dt} \quad \text{Eq. 3-SI}$$

The reaction rates as a function of the substrate concentration can be calculated combining Eqs. 2-SI and 3-SI and can be fitted using the Michaelis-Menten model (Eqs. 6-SI and 7-SI). The derivation of K_M, k_{cat}, K_I, and the α value using ITC requires the knowledge of (i) the total apparent molar enthalpy ΔH_{app} of the reaction under study, and (ii) *dQ/dt* values measured at various substrate concentrations, at the different concentrations of inhibitor tested. Using the inverse single-injection method, ΔH_{app} can be calculated by integrating the area under the curve obtained from the non-inhibited reaction, according to Eq. 4-SI:

$$\Delta H_{app} = \frac{1}{V_{cell} \cdot [S]_{total}} \cdot \int_0^\infty \frac{dQ}{dt} dt \quad \text{Eq. 4-SI}$$

Where $[S]_{total}$ is the total concentration of substrate present in the sample cell at the start of the experiment. By knowing ΔH_{app}, the change in substrate concentration over any time interval can be calculated, at each concentration of inhibitor tested, using Eq. 5-SI:

$$[S]_{t_2} = [S]_{t_1} - \frac{\int_{t_1}^{t_2} \frac{dQ}{dt} dt}{V_{cell} \cdot \Delta H_{app}} \qquad \text{Eq. 5-SI}$$

In this equation, t_1 and t_2 denote two consecutive time points (typically separated by 2 s). Reaction rates as a function of substrate concentration measured at different inhibitor concentrations are then derived using Eq. 3-SI and simultaneously and globally fitted to the Michaelis-Menten equations (Eqs. 6-SI and 7-SI) to determine K_M, k_{cat}, K_I, and α.

For the obtainment of progress curves in the case of tight- and slow-binding inhibitors by ITC, the *TP* traces recorded at increasing concentrations of the tested molecules can be integrated over time (starting from the point at which *TP* reaches its minimum), thus obtaining the total heat (expressed in μcal) associated with the enzyme-catalyzed reaction as a function of the reaction time. The resulting total heat *vs.* time plots can be converted to the final progress curves over time by using the experimentally derived value of ΔH_{app}. In the case of a tight-binding inhibitor, the kinetic parameters v_i can be calculated from the slopes of the progress curves at each inhibitor concentration, and the corresponding K_I^{app} can be determined by using Eq. 8-SI. This value can be converted in the true K_I for a competitive inhibitor by using Eq. 9-SI. In the case of a slow-binding inhibitor, the kinetic parameters v_i and v_s can be calculated from the slopes of the progress curves at each inhibitor concentration, and the corresponding K_I^{app} (K_I^{*app}) and K_I (K_I^{*}) can be determined by using Eqs. 8-SI and 9-SI.

Overview of enzyme kinetics and inhibition

A classical enzyme-catalyzed reaction can be schematized as in Figure 1-SI. The equilibrium between the enzyme (*E*), the substrate (*S*), and the enzyme-substrate (*E•S*) complex is regulated by the kinetic association and dissociation constants k_1 and k_{-1}. The resulting equilibrium dissociation constant K_S is expressed as the ratio k_{-1} / k_1. The most used approach to mathematically describe an enzyme-catalyzed reaction is the Michaelis-Menten model, which expresses the initial reaction rate (*v*), corresponding to the decrease of substrate concentration [*S*] over time, as a function of [*S*], the enzyme concentration [*E*], and the kinetic parameters K_M and k_{cat} (Eq. 6-SI):

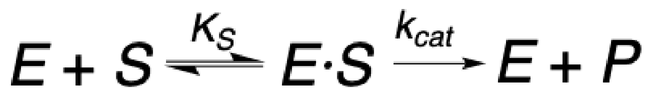

Figure 1-SI. Classical enzymatic reaction scheme.

$$v = -\frac{d[S]}{dt} = \frac{k_{cat}[E][S]}{[S]+K_M} \qquad \text{Eq. 6-SI}$$

In Eq. 6-SI, K_M (commonly known as Michaelis constant) is the pseudo-equilibrium constant [$(k_{-1} + k_{cat})/k_1$] under steady-state conditions, and it represents the substrate concentration required to achieve half-maximal reaction rate. k_{cat} is the catalytic rate constant (also known as turnover number) describing the limiting number of substrate molecules converted per second by the enzyme. The term k_{cat} •[E], also defined as V_{max}, is the maximum reaction rate theoretically achieved in the presence of an infinite amount of substrate. The Michaelis – Menten model can be also used to mathematically describe an enzyme-catalyzed reaction occurring in the presence of a classical reversible inhibitor (Figure 2-SI).

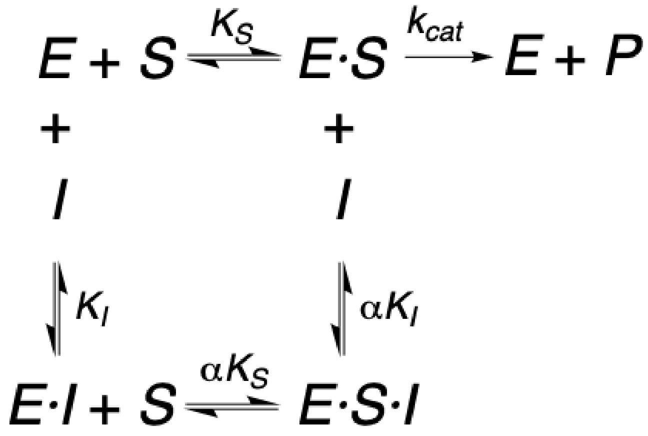

Figure 2-SI. Scheme of an enzymatic reaction inhibited by a classical reversible molecule.

In this case, the complete form of the Michaelis – Menten equation (Eq. 7-SI) includes two additional parameters: i) the equilibrium dissociation constant K_I, commonly known as inhibition constant, and ii) the α value. K_I is in turn described as the ratio of the kinetic dissociation (k_{-3}) and association constants (k_3) that regulate the equilibrium between E, the inhibitor (I), and the enzyme-inhibitor (E•I) complex. The α value provides insight into the mode of inhibition, whether competitive, uncompetitive, or non-competitive, and indicates the degree to which an inhibitor disrupts substrate binding (a higher α denotes a greater competitive contribution to the inhibition)[1, 2].

$$v = -\frac{d[S]}{dt} = \frac{k_{cat}[E][S]}{[S]\left(1+\frac{[I]}{\alpha K_I}\right)+K_M\left(1+\frac{[I]}{K_I}\right)}$$ Eq. 7-SI

The Michaelis-Menten model can be applied for the characterization of enzyme inhibition provided that the inhibitor concentration [*I*] producing a detectable decrease in enzymatic activity greatly exceeds the enzyme concentration [*E*] used in the assay. Under these conditions, the inhibition constant K_I is much larger than [*E*], and the concentration of the enzyme-inhibitor complex [*E•I*] is negligible compared to [I]. Consequently, the free inhibitor concentration can be approximated as $[I]_{free} \approx [I]_{tot}$ throughout the reaction time course. However, some molecules, referred to as tight-binding inhibitors, bind to the target enzyme with such high affinity that significant inhibition is observed at inhibitor concentrations comparable to that of the enzyme. In this case, formation of the *E•I* complex leads to a substantial depletion of free inhibitor, invalidating the Michaelis-Menten assumption that $[I]_{free} \approx [I]_{tot}$. Therefore, a more general approach to characterize enzyme inhibition without *a priori* assumptions regarding the tight- or non-tight-binding behavior relies on progress curves analysis. In this method, product formation [*P*] is monitored as a function of the reaction time at increasing inhibitor concentrations. In the absence of any inhibitor, the enzymatic reaction rate (v_0) remains constant over time, resulting in a linear increase of [*P*] over time, with the slope corresponding to v_0. In the presence of a fast-binding inhibitor, the reaction rate remains constant over time but is reduced, yielding a linear progress curve with a smaller slope (v_i). The reaction rates measured at increasing inhibitor concentration can be used to determine the apparent inhibition constant (K_I^{app}) by using the Morrison's quadratic model[3-5] according to Eq. 8-SI:

$$\frac{v}{v_0} = 1 - \frac{\left([E]+[I]+K_I^{app}\right)-\sqrt{([E]+[I]+K_I^{app})^2-4[E][I]}}{2[E]}$$ Eq. 8-SI

According to Strauss and Goldstein [6, 7], the K_I^{app} / [*E*] ratio discriminates between tight- or non-tight-binding inhibition. When this ratio is greater than 10, ligand depletion by enzyme binding is negligible, such that $[I]_{free} \approx [I]_{tot}$, and classical Michaelis–Menten is applicable. Conversely, when K_I^{app} / [*E*] is less than 10, significant depletion of free inhibitor occurs due to the formation of the *E•I* complex, revealing the tight-binding nature of the inhibitor.

Assuming a competitive inhibition mechanism, the true inhibition constants (K_I) can be derived from K_I^{app} using the Cheng-Prusoff equation [8] (Eq.9-SI):

$$K_I = \frac{K_I^{app}}{1 + \frac{[S]}{K_M}}$$ Eq. 9-SI

The inhibitors described so far (either classical or tight-binding) bind reversibly to enzymes with kinetic association (k_3) and dissociation (k_{-3}) rates, which govern the equilibrium between *E*, *I*, and the ***E•I*** complex, of the same order of magnitude as those (k_1 and k_{-1}) governing the equilibrium between *E*, *S*, and the *E•S* complex. However, some molecules bind reversibly to enzymes with k_3 and k_{-3} that are smaller than k_1 and k_{-1}, in turn establishing their binding equilibrium with the enzyme on a time scale slower than the turnover rate of the enzyme-catalyzed reaction; these ligands are called slow-binding or time-dependent inhibitors. In some cases, slow-binding can occur through a first fast step, with k_{-3} and k_3 of the same order of magnitude as k_1 and k_{-1}, followed by a subsequent slower process, governed by k_4 and k_{-4} constants that are smaller than k_1 and k_{-1}. This latter step is commonly interpreted as a rearrangement of ***E•I*** to a tighter *E•I** complex (Figure 3-SI).

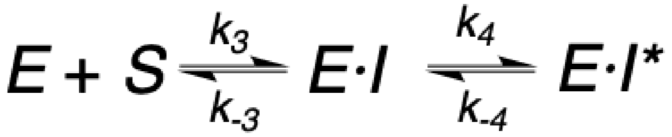

Figure 3-SI. Scheme of an enzymatic reaction inhibited by a slow-binding reversible molecule.

The analysis of slow-binding inhibition also relies on the obtainment of progress curves. However, the enzyme reaction rate in the presence of a slow-binding inhibitor is not constant over time; the resulting progress curves show an initial linear phase, whose slope corresponds to the initial reaction rate (v_i), followed by a transition that eventually evolves toward a second linear phase, with a smaller slope that corresponds to the steady-state (v_s) rate. The steady-state rate is reached after the equilibrium between *E*, *I*, and the ***E•I*** complex (or the *E•I** complex in the case of a two-step inhibition) has been established. In the case of a tight- and slow-binding inhibitor, the sets of v_i and v_s values measured at the different inhibitor concentrations can be used separately to quantitatively derive the apparent inhibition constants (K_I^{app} and K_I^{*app}) for these two steps by using Eq. 8-SI. Assuming a competitive inhibition mechanism, the true inhibition constants (K_I and K_I^*) can be again derived using Eq. 9-SI.

Figure 4-SI. Baseline-uncorrected raw calorimetric traces of the 3CL^{pro} kinetic experiments carried out using the progress-curves approach in the presence of ML300 (A), X77 (B), and Nirmatrelvir (C), and the Michaelis-Menten approach in the presence of ML300 (D) and X77 (E).

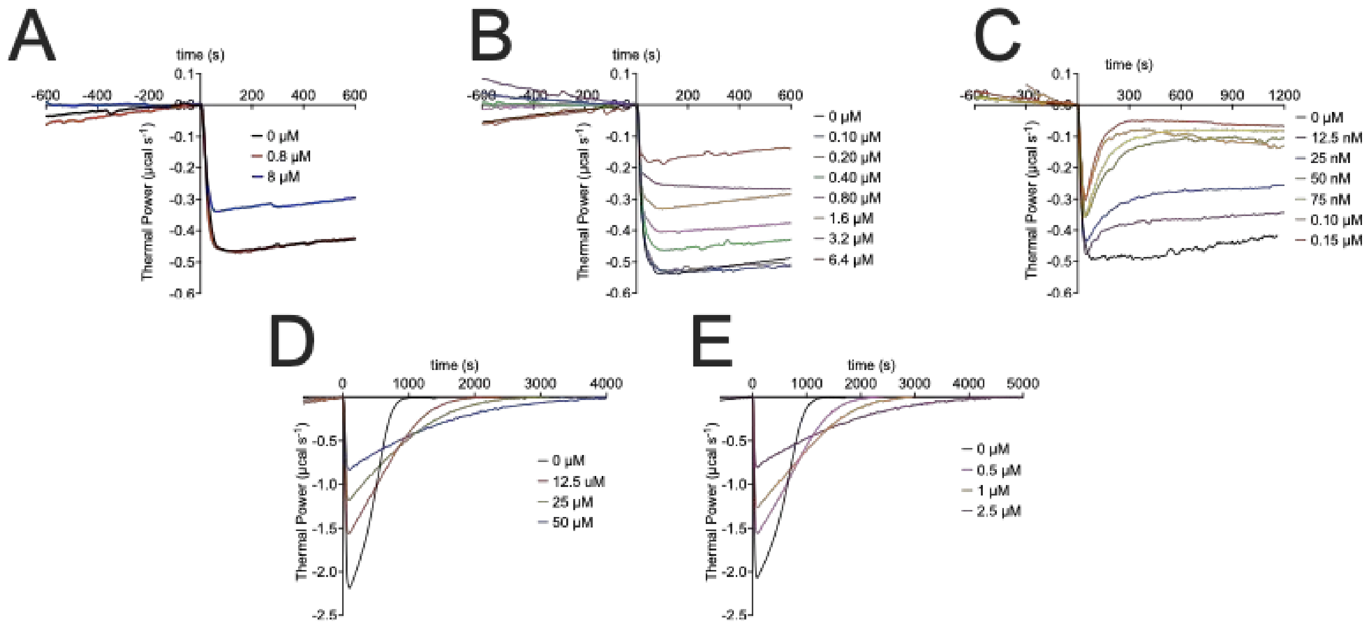

Figure 5-SI. Baseline-uncorrected raw calorimetric traces of the 3CL^{pro} (wild-type, C145A single mutant, and E290A/R298A double mutant) binding experiments to Ensitrelvir (ENS), ML300, X77, and Nirmatrelvir (NMV). Plots marked with an * refer to the binding experiments carried out at lower concentration of wild-type 3CL^{pro} to Ensitrelvir and Nirmatrelvir.

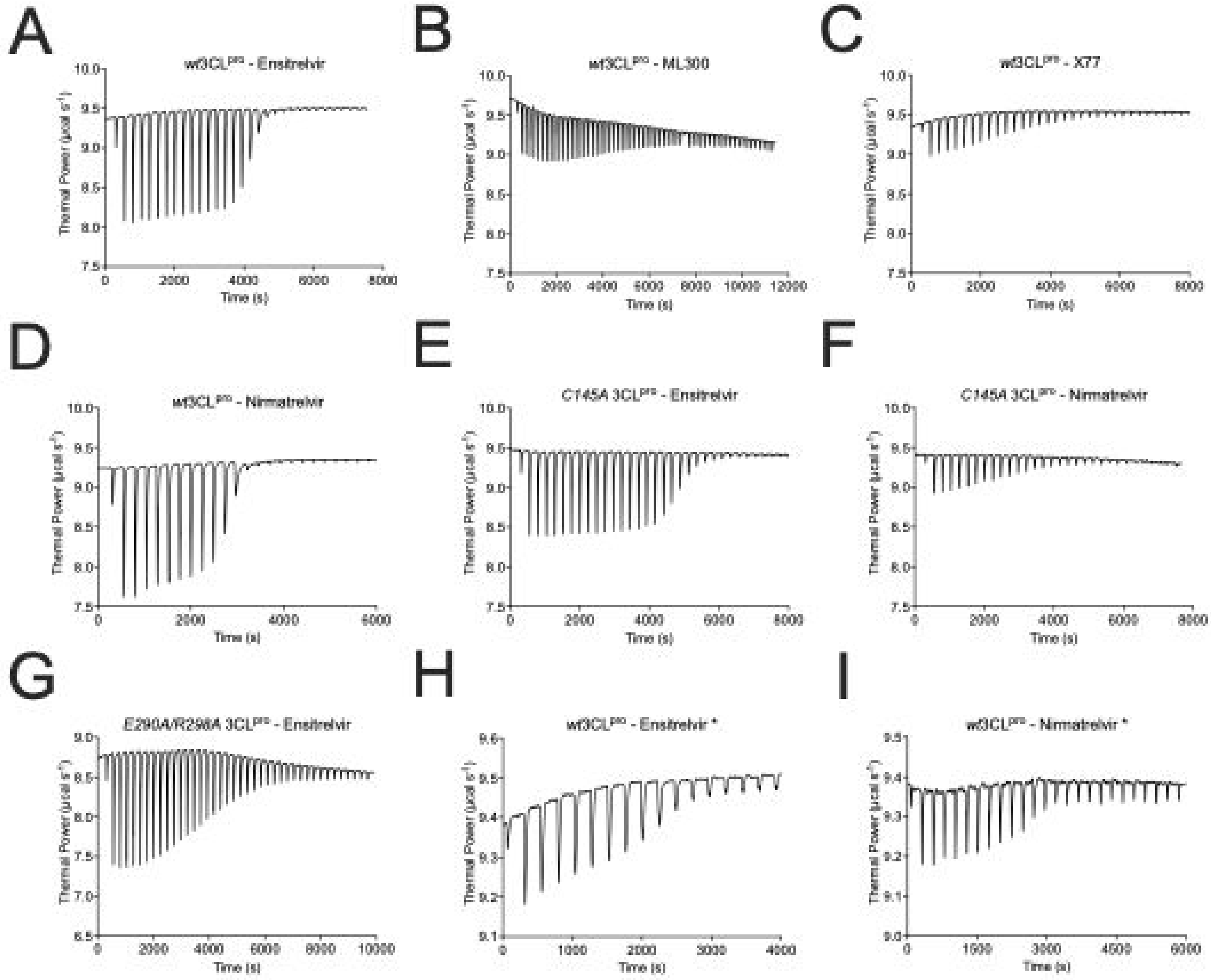

Figure 6-SI. Representative titrations of 5 µM wild type 3CL^{pro} with 150 µM Ensitrelvir and 3 µM wild type 3CL^{pro} with 50 µM Nirmatrelvir. (A,B) Heat response for injections of 150 µM Ensitrelvir (A) and 50 µM Nirmatrelvir (B) onto 3CL^{pro}. (C,D) Integrated heats *vs.* molar ratio with best-fit single-site isotherms (red lines). Dissociation constants are also shown as determined using either the Origin 7.0 software with their corresponding standard errors ([a]), or ACI-ITC with the 95 % confidence interval in brackets ([b]).

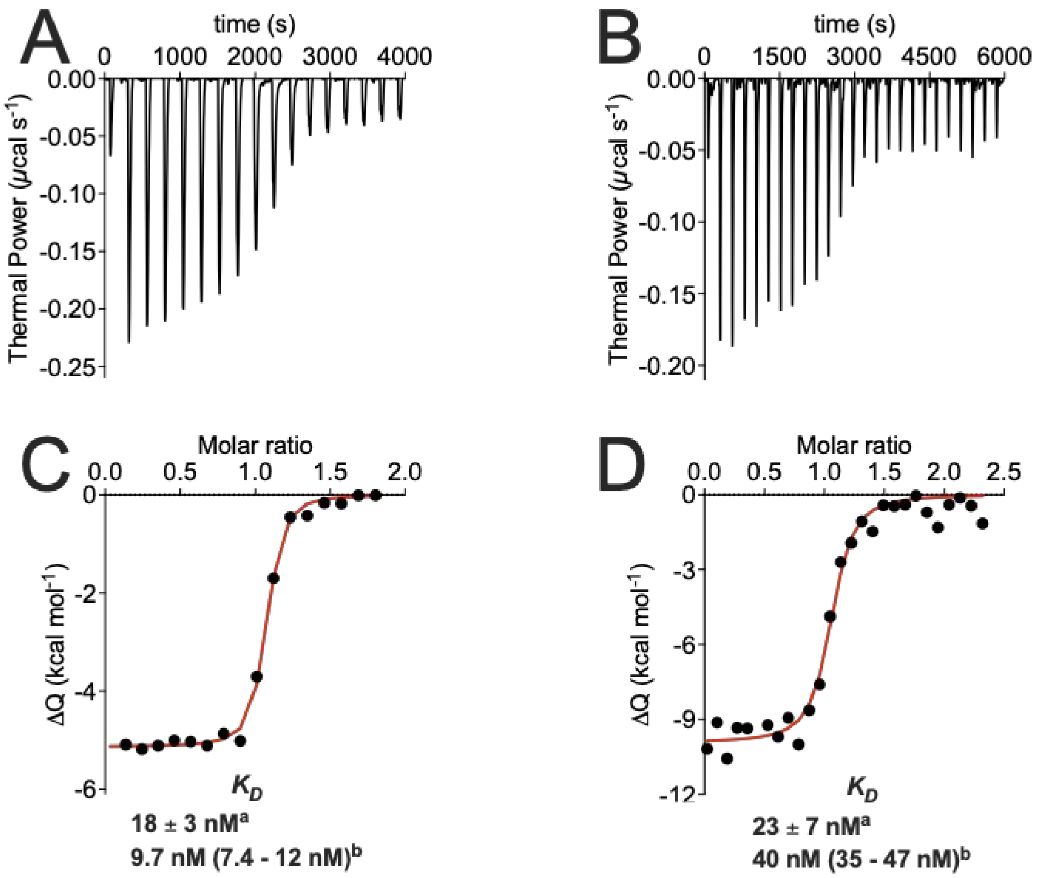

Table 1-SI. Thermodynamic parameters derived from ITC binding experiments.

	n	K_B (M^{-1})	*ΔH* (kcal mol^{-1})	*ΔS* (cal mol^{-1} K^{-1})	*ΔG* (kcal mol^{-1})
*wt*3CL^{pro}-ENS	1.0 ± 0.1	(1.9 ± 0.2) x 10^{-8}	-5.1 ± 0.1	18.2	-10.6
*wt*3CL^{pro}-ML300	1.1 ± 0.1	(7.5 ± 0.4) x 10^{-6}	-9.6 ± 0.2	-8.6	-7.0
*wt*3CL^{pro}-X77	1.0 ± 0.1	(2.8 ± 0.3) x 10^{-6}	-6.8 ± 0.1	2.5	-7.6
*wt*3CL^{pro}-NMV	0.9 ± 0.1	(1.5 ± 0.2) x 10^{-8}	-5.5 ± 0.1	17.1	-10.6
C145A 3CL^{pro}-ENS	1.0 ± 0.1	(1.5 ± 0.2) x 10^{-7}	-14.1 ± 0.1	-16.0	-9.3
C145A 3CL^{pro}-NMV	1.0 ± 0.1	(4.4 ± 0.8) x 10^{-6}	-6.3 ± 0.3	3.4	-7.3
E290A/R29A 3CL^{pro}-ENS	1.0 ± 0.1	(3.8 ± 0.2) x 10^{-6}	-20.3 ± 0.2	-43.4	-7.4

ACCESSION CODES

3C-like proteinase nsp5 (3CL^{pro})

UniProt entry: P0DTD1

UniProt chain ID: PRO_0000449623

NCBI: YP_009725301.1

REFERENCES

1. Copeland, R. A., Reversible Modes of Inhibitor Interactions with Enzymes. *Evaluation of Enzyme Inhibitors in Drug Discovery* **2013**, 57-121.
2. Copeland, R. A., Reversible Inhibitors. *Enzymes* **2000**, 266-304.
3. Morrison, J. F., Kinetics of the reversible inhibition of enzyme-catalysed reactions by tight-binding inhibitors. *Biochimica et Biophysica Acta (BBA) - Enzymology* **1969,** *185* (2), 269-286.
4. Williams, J. W.; Morrison, J. F., The kinetics of reversible tight-binding inhibition. *Methods in Enzymology* **1979,** *63*, 437-467.
5. Copeland, R. A., Tight Binding Inhibition. *Evaluation of Enzyme Inhibitors in Drug Discovery* **2013**, 245-285.
6. Straus, O. H.; Goldstein, A., Zone Behavior of Enzymes: Illustrated by the Effect of Dissociation Constant and Dilution on the System Cholinesterase-Physostigmine. *J. Gen. Physiol.* **1943,** *26*, 559-585.
7. Copeland, R. A., Evaluation of enzyme inhibitors in drug discovery. A guide for medicinal chemists and pharmacologists. *Methods Biochem. Anal.* **2005,** *46*, 1 - 265.
8. Copeland, A., Tight Binding Inhibitors. In *Enzymes*, 2000; pp 305-317.
